# Methods and Effectiveness of Auditory-Cognitive Training for Older Adults: Scoping Review and Meta-Analysis

**DOI:** 10.2196/101632

**Published:** 2026-11-09

**Authors:** Mengyuan Li, Chifen Ma, Piers Dawes, Yanni Yang, Weiwei Liu

**Affiliations:** 1 School of Nursing, Army Medical University, No.30 Gaotanyanzhengjie Street, Shapingba District, Chongqing, 400038, China, 86 13370122249; 2 College of Health Services and Management, Xuzhou, China; 3 University of Queensland Centre for Hearing Research (CHEAR), School of Health and Rehabilitation Sciences, The University of Queensland, Brisbane, Queensland, Australia

**Keywords:** hearing loss, auditory-cognitive training, older adults, cognitive decline, auditory training

## Abstract

**Background:**

Auditory-cognitive training (ACT) combines auditory and cognitive exercises to improve listening and cognition in individuals with hearing difficulties; yet, it lacks standardized implementation and consistent evidence.

**Objective:**

This mixed-evidence synthesis aimed to map ACT’s implementation characteristics and summarize its reported short-term and maintenance effects on trained and nontrained cognitive and auditory outcomes, including hearing function in daily life.

**Methods:**

The review followed the PRISMA-ScR (Preferred Reporting Items for Systematic Reviews and Meta-Analyses extension for Scoping Reviews) guidelines and the Arksey and O’Malley scoping review framework. Databases searched included Embase, the Cochrane Library, PubMed, and APA PsycNet from inception to July 30, 2026. Articles were included or excluded based on specific criteria, and included articles were evaluated for risk of bias.

**Results:**

ACT showed significant pooled effects on speech-in-noise performance (mean difference [MD]=−1.23 dB, 95% CI −2.35 to −0.10; *P*=.03), attention (standardized mean difference [SMD]=0.50, 95% CI 0.23-0.77; *P*<.001), memory (SMD=1.08, 95% CI 0.25-1.92; *P*=.01), and global cognition (MD=3.27, 95% CI 0.27-6.26; *P*=.03). Effects on pure-tone audiometry, subjective hearing function, working memory, and executive function were limited and nonsignificant. Current evidence is limited by unstandardized training dosage/duration, limited linguistic generalizability across non-English languages, and insufficient assessment of patient-reported real-life listening outcomes.

**Conclusions:**

ACT may improve selected auditory and cognitive outcomes in older adults, particularly speech-in-noise perception, and attention. The evidence remains limited by substantial heterogeneity, a small number of studies, and low certainty for most outcomes. Standardized protocols, clinically meaningful outcome measures, and longer follow-up are needed to determine its clinical effectiveness and long-term benefits.

## Introduction

Hearing function is critical for daily social connections and often declines with age. Globally, 1.57 billion people have hearing loss (HL), and this number is projected to reach 2.17 billion by 2040 [1]. In China, over 70% of older adults are affected [2]. Beyond auditory impairment, age-related HL is a modifiable risk factor for adverse outcomes in older adults, including accelerated cognitive decline, dementia, social isolation, depression, and reduced quality of life [3-6]. Severe HL increases dementia risk 5-fold compared to normal hearing [7].

Hearing aids (HAs) are the primary treatment; however, only 7.7% of patients with HL use them [8], mainly due to persistent speech-in-noise (SIN) difficulties [9]. SIN perception relies on auditory and cognitive functions (eg, attention and working memory), which synergistically affect listening in older adults [9,10]. This has shifted interventions from “bottom-up” auditory training to integrated auditory-cognitive training (ACT) [11].

The theoretical underpinnings of ACT are rooted in neuroplasticity and the Ease of Language Understanding (ELU) model. Neuroplasticity theory suggests that ACT induces neural remodeling to enhance auditory-cognitive coordination [12], while the ELU model highlights working memory’s role in resolving ambiguous auditory input—challenging for older adults [13]. Unlike HAs, which only amplify sound, ACT addresses cognitive deficits underlying SIN perception.

Preliminary ACT studies in older adults with HL show dual benefits for SIN perception [14-17] and cognitive functions (eg, memory, attention, and processing speed) [14,15,18]. However, findings are heterogeneous due to variable study designs, interventions, and outcomes, and the magnitude and consistency of the effects are unclear.

No consensus has yet been established regarding the optimal implementation of ACT. Existing studies vary considerably in terms of training domains, intervention dosage, and delivery modalities. For example, training duration ranges widely, from as short as 2 weeks [17] to as long as 8 weeks [19]. The targeted training domains are heterogeneous: some studies focus on auditory or speech perception and recognition [14,18], whereas others adopt integrated approaches combining auditory training with cognitive components such as attention, working memory, or executive function [20]. Such variability hinders the quantification of effect sizes, limits cross-study comparisons, and impedes the development of standardized, replicable ACT protocols.

Thus, this study systematically maps ACT implementation characteristics (modalities, dosage, and participants’ hearing status) and quantitatively synthesizes its effects on auditory and cognitive outcomes via meta-analysis. It aims to provide a comprehensive overview of current ACT interventions and evidence-based guidance for future design and clinical applications.

This review aimed to map existing literature and characterize ACT interventions for older adults with or without HL. It synthesized ACT implementation characteristics and summarized evidence for effects on hearing and cognitive outcomes. Four research questions guided the review:

1. What are the hearing and cognitive profiles of older adults for whom ACT is applicable in existing studies?
2. How should ACT be designed and delivered (eg, content, modality, and difficulty adjustment) for older adults?
3. What is the evidence for the effects of ACT on hearing and cognitive outcomes, and what are the pooled effect sizes from meta-analyses?
4. What key limitations and research gaps exist in the current ACT literature?

## Methods

### Overview

This review followed the PRISMA (Preferred Reporting Items for Systematic Reviews and Meta-Analyses) 2020 guidelines, PRISMA-ScR (Preferred Reporting Items for Systematic Reviews and Meta-Analyses extension for Scoping Reviews) guidelines, and the Arksey and O’Malley scoping review framework [21-23]. The protocol, including synthesis methods, was reregistered on Open Science Framework (OSF) before data collection [24]. The PRISMA 2020 and PRISMA-ScR checklists are provided in Checklists 1 and 2 [25].

### Search Strategy

A systematic literature search was conducted in Embase, the Cochrane Library, PubMed, and APA PsycNet, supplemented by manual screening of the reference lists of included studies and relevant reviews. The core search concepts included older adults, HL, auditory training, and cognitive training, with database-specific subject headings, keywords, Boolean operators, and search syntax adapted for each database. No restrictions on study design were applied during the initial search. Studies published in English and available through the final search date of July 30, 2026, were considered eligible. We also manually screened the reference lists of the included studies and relevant reviews to identify potentially eligible studies that may not have been retrieved through the database searches. The date of each database search and the complete database-specific search strategies, including all search terms, field specifications, and database-specific operators, are provided in Multimedia Appendix 1.

### Eligibility Criteria

Studies were eligible for inclusion in the scoping review if they met the following criteria: participants were older adults, with or without HL. The review focused on ACT interventions, defined as those that explicitly integrate auditory processing training with cognitive components (eg, attention, working memory, executive function, or cognitive control) and have the primary aim of improving speech perception, auditory processing, or communication abilities in complex acoustic environments. No restrictions were applied regarding study design. Studies published in English before August 2026 were considered eligible.

For the subsequent meta-analysis, eligibility criteria were further refined. Only randomized controlled trials (RCTs) and quasi-experimental studies were included. Eligible studies were required to compare ACT interventions with a control condition, including education-based controls, waitlist controls, usual care, or traditional auditory rehabilitation. Studies were included if they reported quantitative outcomes related to auditory function (eg, speech perception, SIN performance, and auditory processing) and/or cognitive function (eg, attention, executive function, working memory, or global cognition), and provided sufficient data for effect size calculation (eg, means, SDs, and sample sizes).

Studies were excluded if they did not involve an explicit integrative auditory-cognitive intervention, focused solely on auditory training or on cognitive training without integration, lacked full-text availability (eg, protocols and conference abstracts), or did not provide sufficient data for meta-analysis.

### Extracting and Charting the Data

Data were extracted independently by 2 reviewers (ML and CM) using a standardized form, with discrepancies resolved by consensus. Extracted data included study characteristics, participant demographics, hearing and cognitive status, ACT design, delivery mode, dosage, outcome measures, and key results. These data were synthesized descriptively to provide a comprehensive overview of the characteristics, intervention designs, and outcomes of ACT studies in older adults.

### Quality of Evidence

Two reviewers independently assessed the methodological quality of the included studies. For RCTs, the revised Cochrane risk-of-bias tool (RoB 2) [26] was used to evaluate bias across 5 domains: randomization process, deviations from intended interventions, missing outcome data, measurement of outcomes, and selection of the reported result. For quasi-experimental studies, the Joanna Briggs Institute (JBI) critical appraisal checklist for quasi-experimental studies was applied [27]. Disagreements between reviewers were resolved through discussion or consultation with a third reviewer. Among the 24 included publications, 3 articles reported the same underlying study populations as other publications and were considered multiple reports of the same study. Therefore, risk-of-bias assessment was conducted at the study level, including 16 independent RCTs assessed using RoB 2 and 5 quasi-experimental studies assessed using the JBI checklist.

### Synthesis Methods

The synthesis methods were preregistered before data collection on the OSF [24]. Findings were integrated through qualitative and quantitative synthesis. Content analysis was used to summarize nonquantitative findings, and a Sankey diagram was used to visualize outcome patterns. Meta-analyses were conducted in R (version 4.4.2; R Foundation for Statistical Computing) using the meta package. For continuous outcomes, intervention effects were calculated using change scores from baseline to postintervention. Mean differences (MDs) were used when outcomes were measured on the same scale, whereas standardized mean differences (SMDs; Hedges *g*) were used when the same construct was assessed using different scales. When change-score SDs were not directly reported, they were calculated from baseline and postintervention SDs assuming a correlation of 0.50. For studies reporting within-group change scores and Cohen *d* but not change-score SDs, the SDs were derived from the reported mean changes and Cohen *d* and were incorporated into the corresponding meta-analyses. Fixed-effects models were applied when heterogeneity was low (*I*²<50%), and random-effects models were applied when heterogeneity was moderate to high (*I*²≥50%) [28]. Heterogeneity was assessed using Cochran *Q* test and *I*². Forest plots were used to present pooled effect estimates and 95% CIs; the Egger test and funnel plots were used to assess potential publication bias when sufficient studies (≥10) were available.

## Results

### Overview

A total of 688 records were identified. After removing 43 duplicates, 645 records were screened, of which 586 were excluded based on title and abstract. A full-text evaluation of the remaining 59 articles resulted in a further 35 exclusions (non-ACT interventions, reviews, unavailable texts, non-English papers, and single-modal auditory training), yielding 24 eligible articles. Among these, 3 articles were identified as multiple reports of the same intervention study, involving the same participants, group allocation, and intervention protocol. Therefore, the 24 included articles represented 21 independent studies. Eight studies were excluded from the meta-analysis for lack of data. Therefore, 16 reports corresponding to 14 studies were included in the meta-analysis. The screening process is shown in Figure 1.

The 21 included studies were published between 2007 and 2026, with 9 published in the past 5 years. Eleven were conducted in the United States, 2 in the United Kingdom, and 1 each in Switzerland, Japan, Singapore, India, Germany, Turkey, China, and Malaysia. Sixteen studies used RCT designs, and 5 used quasi-experimental designs. Sample sizes of intervention groups ranged from 11 to 63, and participant ages varied from 40 to 89 years. Participant demographic characteristics are summarized in Table 1.

**Figure 1.**
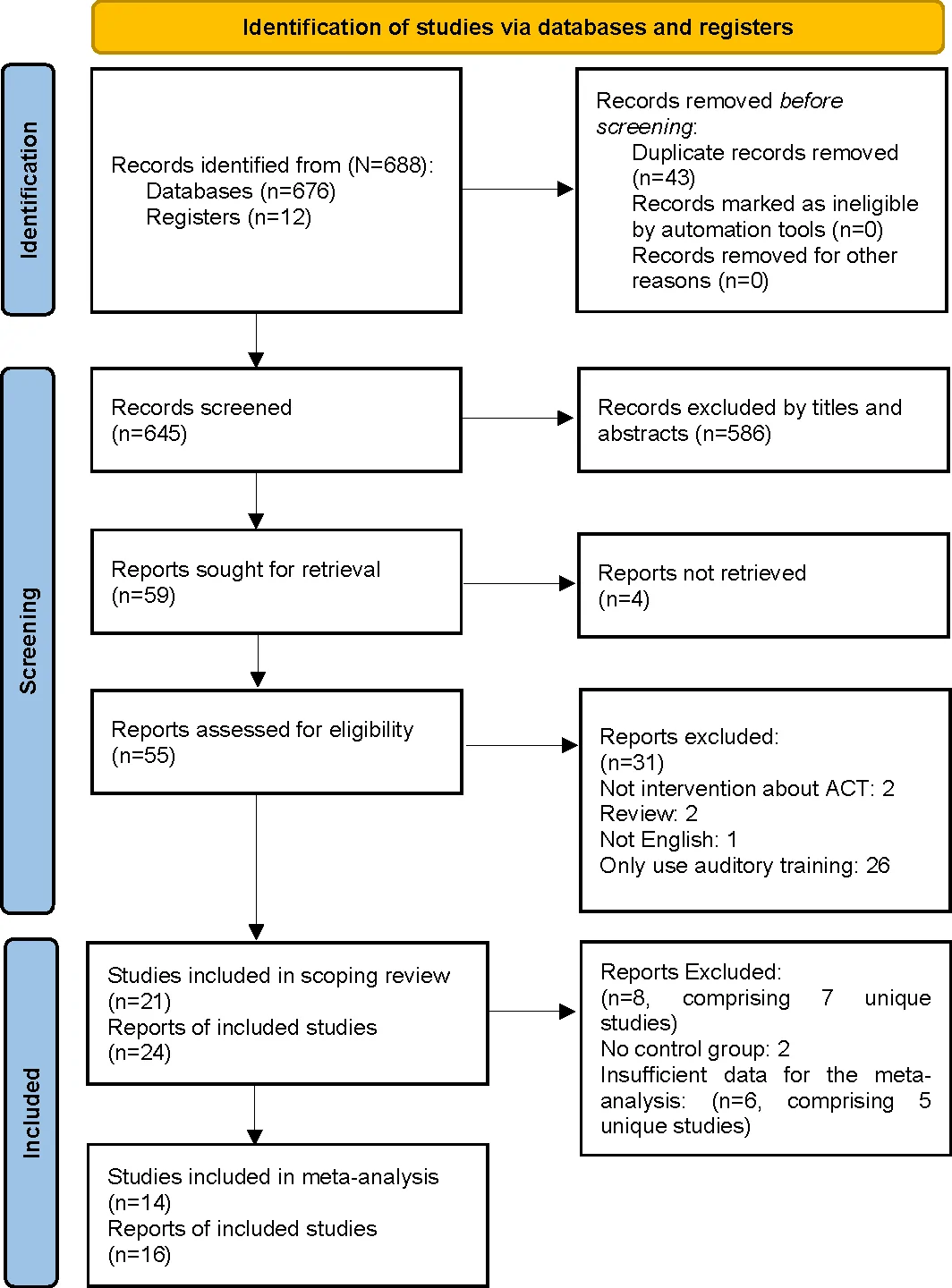
PRISMA (Preferred Reporting Items for Systematic Reviews and Meta-Analyses) flowchart. ACT: auditory-cognitive training.

**Table 1.** Demographic characteristics of included studies.

Study ID	Country	Study design	Sample size (n)		Age (years)	Gender	Hearing status	Cognition status
			T group^a^	Ctrl group^b^				
Abrams et al [29], 2015	United States	RCT^c^	15	14	T group: mean 65.6 (SD 5.53); Ctrl group: mean 61.8 (SD 8.4)	T group: 11 men, 4 women; Ctrl group: 8 men, 6 women	Bilateral symmetrical sensorineural HL^d^; include new and experienced HA^e^ users	Not reported
Anderson et al [14], 2013	United States	RCT	29	29	Mean 64.11 (SD 5.78)	Women: 35, men: 23	Normal hearing: PTA^f^ (6 Hz) <25 dB (n=28); HL: PTA (8 Hz) <80 dB (n=30); no HA users	Normal IQ, without neurologic conditions
Anderson et al [15], 2013	United States	RCT	35	32	Mean 63.0 (SD 3.7)	Women: 38, men: 29	PTA (4 kHz) ≤45 dB; no HA users	Normal IQ, MoCA^g^≥23
Faust et al [18], 2020	United States	RCT	37	37	Range: 68.0-79.8	Not reported	No reported HL; no HA users	MMSE^h^≥24
Fisher et al [16,30], 2025	United States	RCT with pseudo-randomization	Low cognitive group: 25; high cognitive group: 12	None	Range: 60-83	Women: 19, men: 4	Normal/near-normal: PTA (250‐4000 Hz) ≤25 dB HL (both ears); no HA users	MoCA≥26
Frei and Giroud [17], 2025	Switzerland	RCT, crossover design	39	None	Mean 70 (SD 4.92)	Women: 19, men: 20	PTA (0.5, 1, 2, 4 kHz) ≤40 dB; no HA users	No preexisting neurological diseases, cognition not reported
Frost et al [31], 2025	United Kingdom	RCT	22	21	Range: 40-75	Women: 28, men: 15	Mild subjective hearing concern; objective: normal/mild-to-moderate HL; no HA users	Mild subjective memory concern, without dementia diagnosis, not suspected to have moderate-to-severe hearing or cognitive impairment
Ferguson et al [32], 2014	United Kingdom	RCT, crossover design	23	DT^i^ group: 21	Range: 50-74	Women: 15, men: 29	Symmetrical mild sensorineural HL: PTA (0.5‐4 kHz, better ear) 21‐40 dB HL; no HA users	Not reported
Gundmi et al [33], 2026	India	RCT	40	40	Range: 45‐65	Women: 34, men: 46	Bilateral mild-to-moderate sensorineural HL (PTA 26‐70 dB HL); all HA users	MMSE≥24
Henderson Sabes [34], 2007; Sweetow and Henderson Sabes [35], 2006	United States	RCT, crossover design	38	27	Mean 64.7 (SD 14.9)	Not reported	56 with HL; 9 with speech understanding difficulties (5 had normal PTA); HA users: n=56	Not reported
Kawata et al [20], 2022	Japan	RCT	AC^j^: 13; auditory: 13; cognitive: 14	12	Mean 68.07 (SD 4.14)	Women: 43, men: 14	Normal-to-mild HL; no HA users	MoCA≥26
Kucuk Ceyhan et al [36], 2022	Turkey	Parallel-group quasi trial	17	16	T group: mean 72.17 (SD 6.94); Ctrl group: mean 71.75 (SD 6.81)	T group: 8 men, 9 women; Ctrl group: 10 men, 6 women	Age-related bilateral symmetrical sensorineural HL, thresholds ≤40 dB HL at 500 Hz, ≤80 dB HL at 4000 Hz; all HA users	MMSE≥24
Lai et al [37], 2023	Singapore	Three-group quota-sampled parallel trial	20	Active: 21; no-training: 23	Experimental: mean 66.7 (SD 4.55); active Ctrl: mean 66.2 (SD 5.79); no-training Ctrl: mean 67.26 (SD 6.11); all aged 60‐87	Not reported	Mild-to-moderate age-related HL, 4 frequency PTA average 30‐34 dB HL; no HA users	MMSE≥25
Ockelmann et al [38], 2025	Germany	RCT	27	27	Range: 65-82	Women: 30, men: 30	Mild-to-moderate HL: PTA 20‐60 dB; HA users: n=21	MoCA≥23
Olson et al [39], 2013	United States	Prospective repeated-measures quasi-experiment	14	7	Overall: 66‐68; new training, mean 66 (SD 9.6); experienced, mean 68 (SD 6.9); new Ctrl, mean 66 (SD 9.3)	New training: 1 man, 7 women; experienced: 10 men, 4 women; new control: 7 men	Mild-to-severe sensorineural HL; include new and experienced HA users	Not reported
Rao et al [40], 2017	United States	Parallel-group quasi-experiment	11	11	T group: range: 60‐85, mean 69 (SD 7.3); Ctrl group: range: 49‐85, mean 65 (SD 8.9)	T group: 6 men, 5 women; Ctrl group: 6 men, 5 women	Mild-to-moderate bilateral sensorineural HL; thresholds ≤40 dB≤1500 Hz, ≤70 dB at high frequencies; all new HA users	MMSE=27‐30
Rishiq et al [41], 2016	United States	RCT	12	12	T group: range; 62‐81; Ctrl group: range: 51‐84	T group: 6 men, 6 women; Ctrl group: 8 men, 4 women	Mild-to-moderate high-frequency sensorineural HL; all HA users	MMSE=27‐30
Saunders et al [42], 2016; Smith et al [43], 2016	United States	RCT	LACE-C^k^: 61; LACE-DVD: 63	Placebo audiobook training: 69; educational counseling: 70	Mean 68.6 (SD 7.7)	Not reported	Symmetrical mild-to-moderate sensorineural HL; all HA users	Age and educationally appropriate scores on MMSE
Whitton et al [44], 2017	United States	RCT	13	11	Range: 50‐89, mean 70	Women: 32, men: 16	Mild-to-severe bilateral sensorineural HL; all long-term users	MoCA>25, WASI^l^ full IQ>80, GDS^m^<6
Yusof et al [19], 2019	Malaysia	Quasi-experiment	40	36	Range: ≥60	Women: 57, men: 19	Symmetrical: PTA (0.5‐4.0 kHz, better ear) ≤40 dB HL; no HA users	NC^n^ and NCI^o^ group
Zhao et al [45], 2026	China	Pilot RCT	30	30	Mean 67.65 (SD 4.78)	Women: 15, men: 45	Mild-to-moderate age-related HL, PTA 20‐50 dB; no HA users	MoCA≥26

a T group: training group.

b Ctrl group: control group.

c RCT: randomized controlled trial.

d HL: hearing loss.

e HA: hearing aid.

f PTA: pure-tone average.

g MoCA: Montreal Cognitive Assessment.

h MMSE: Mini-Mental State Examination.

i DT: delay training.

j AC: auditory-cognitive.

k LACE: listening and communication enhancement.

l WASI: Wechsler’s Abbreviated Scale of Intelligence.

m GDS: Geriatric Depression Scale.

n NC: normal cognition.

o NCI: neurocognitive impairment.

### Quality of Evidence

Risk-of-bias assessments are presented in Multimedia Appendix 2. Among the 16 RCTs assessed using RoB 2, overall risk of bias was rated as low risk in 18.8% (n=3) of studies, some concerns in 75% (n=12), and high risk in 6.3% (n=1). The majority of concerns were related to the randomization process and selection of the reported results. Specifically, 31.3% (n=5) of studies were judged as having low risk, 62.5% (n=10) as having some concerns, and 6.3% (n=1) as having high risk regarding the randomization process. For deviations from intended interventions, most studies were rated as low risk (n=14, 87.5%). All studies showed low risk for the measurement of the outcome domain, whereas most studies had some concerns regarding selection of the reported results (n=13, 81.3%).

For the 5 quasi-experimental studies assessed using the JBI checklist, most studies demonstrated adequate methodological quality. However, several studies had potential concerns regarding selection and allocation procedures, temporal precedence, and control of confounding factors. Detailed assessments for each domain are presented in Multimedia Appendix 2 (JBI critical appraisal qualitative evidence syntheses).

### Participants’ Hearing and Cognitive Characteristics

Hearing characteristics were heterogeneous across studies. Four studies included participants with normal or near-normal hearing, 7 studies focused on mild HL, and 9 studies investigated mild-to-moderate HL. Five studies included mixed hearing profiles, combining participants with different degrees of hearing ability. Because several studies recruited heterogeneous samples, some studies were classified into multiple categories. HA use was uncommon; only 2 studies included HA users, whereas most studies enrolled nonusers.

Twelve studies included cognitively normal older adults, defined via cutoffs on Mini-Mental State Examination (MMSE; MMSE scores: ≥24‐25 or between 27 and 30)/Montreal Cognitive Assessment (MoCA; MoCA scores: ≥26 or ≥23) or IQ testing and a clinical interview (n=2; normal IQ). One study enrolled participants with educationally appropriate scores on the MMSE, one enrolled participants with subjective cognitive decline, and one included both cognitively normal and mild cognitive impairment groups. The remaining 4 studies did not report sufficient cognitive information.

### ACT Summary

Table 2 provides an overview of ACT delivery methods, modalities, content, and dosage across the included studies; detailed intervention and control characteristics, follow-up, and measurements are provided in Table S1, Multimedia Appendix 1.

**Table 2.** Summary of auditory-cognitive training intervention characteristics^a^.

Domain	Summary of specific characteristics	
Training system	Commercial integrated cognitive training systems, including Brain Fitness, Insight, CogniFit, ActivEar, and ReadMyQuips Commercial auditory rehabilitation system: LACE^b^ AI Pro and LACE DVD/online Researcher-developed training systems, including APEX^c^ 4, HELIX^d^, Closed-Loop, and other customized platforms	
Training content	Pure auditory training: tone discrimination, phoneme discrimination, syllable sequence recall, speech comprehension, sound source localization, and speech recognition under noise-masking conditions Auditory-cognitive integrated training: auditory processing speed training, instruction-following tasks, adaptive auditory memory training, word or sentence recall (short-term memory), backward digit recall (working memory), audio-based vocabulary span training, and Go/No-Go tasks (attention) Cognitive training: tracking moving targets, spatial memory tasks, rapid motion direction identification, and word memory training	
Training delivery modality	Computer-based training (14 studies), mobile or tablet-based apps (4 studies), immersive virtual reality–based training (1 study), and one-to-one supervised training (2 studies)	

a Training frequency and dosage characteristics were as follows: training period, median 4 (IQR 1-12) weeks; sessions per week, median 5 (IQR 1-7) sessions; session duration, median 30 (IQR 10-60) minutes; total number of sessions, median 20 (IQR 2-60) sessions; and total training duration, median 10 (IQR 1.5-60) hours.

b LACE: listening and communication enhancement*.*

c APEX: Audiological Platform for Experimental Research.

d HELIX: Hearing and Listening Experience.

#### Training Methods and Delivery Modalities

Fourteen studies used computer-based training; 4 used mobile app–based training; 1 used immersive virtual reality (VR); and 2 used face-to-face supervised training. Training tools included 5 commercial cognitive platforms (Brain Fitness [Posit Science Corp], Insight [Posit Science Corp], CogniFit [CogniFit Inc], ActivEar [CogniFit Inc], and ReadMyQuips [Sense Synergy, Inc]), a commercial auditory rehabilitation program (LACE AI Pro [Neurotone]), and researcher-developed systems (eg, APEX [Audiological Platform for Experimental Research] 4, HELIX [Hearing and Listening Experience], and Closed-Loop). Notably, all the training systems featured adaptive difficulty for individualized training.

#### Training Content

With respect to training content, ACT interventions were classified into auditory training, auditory-cognitive combined training, and cognitive training. Auditory training targeted auditory perception and speech processing, including tone and phoneme discrimination, syllable sequence recall, speech comprehension, sound source localization, and speech recognition under noise-masking conditions. Auditory-cognitive training integrated auditory tasks with cognitive demands, such as auditory processing speed training, instruction-following tasks, adaptive auditory memory exercises, word or sentence recall targeting short-term memory, backward digit recall tasks engaging working memory, audio-based vocabulary span training, and attention-related paradigms such as Go/No-Go tasks. Cognitive training components included tracking moving targets, spatial memory tasks, rapid motion direction identification, and word memory training.

#### Training Dosage and Intensity

The median training period was 4 (IQR 1-12) weeks. The median weekly frequency was 5 (IQR 1-7) sessions, and the median session duration was 30 (IQR 10-60) minutes. The median number of total sessions was 20 (IQR 2-60) sessions, and the median cumulative training time was 10 (IQR 1.5-60) hours.

### ACT Training Outcomes Summary

#### Overview

Figure 2 summarizes the main outcomes, task effect categories, short-term and maintenance effects, and corresponding intervention effectiveness. A narrative summary of key findings, task effects, time effects, and auditory/cognitive/neural outcomes is presented in Table S2, Multimedia Appendix 1. The detailed narrative summary of each outcome, including the studies assessing short-term and follow-up effects, task-effect classification (on-task: the outcome was trained during the intervention; off-task: the outcome was not trained during the intervention), and the effectiveness of ACT in the intervention group, is provided in Multimedia Appendix 3.

Overall, off-task, nontrained outcomes (15 studies) were assessed more frequently than on-task, trained outcomes (6 studies). Among the auditory-related outcomes, more than half of the short-term effects improved significantly, and half of the maintenance effects were significant. For the cognitive-related outcomes, almost half of the short-term effects were significant, but only one-third of the maintenance effects were significant.

**Figure 2.**
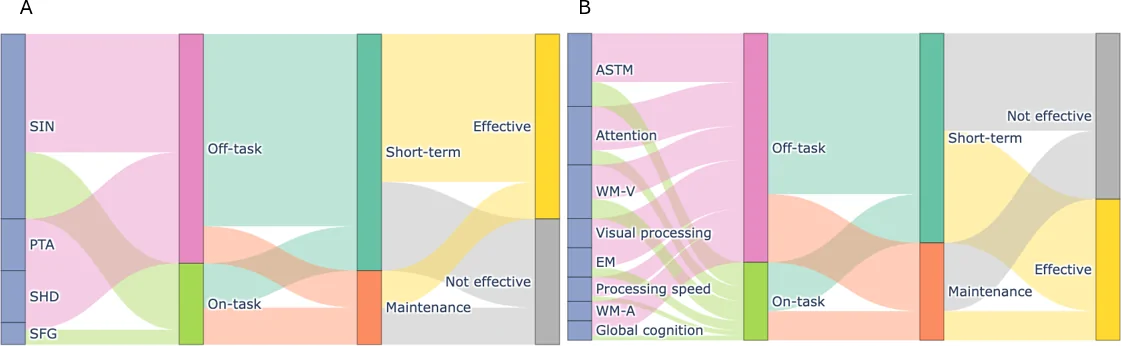
(A) Auditory-related outcomes and (B) cognitive-related outcomes: auditory and cognitive effects, task attributes, and effectiveness. ASTM: auditory short-term memory; EM: episodic memory; HE: hearing effort; PTA: pure-tone average; SFG: auditory stream segregation; SHD: self-report hearing difficulty; SIN: speech-in-noise; WM-A: working memory (auditory); WM-V: working memory (visual).

#### Auditory-Related Outcomes

Among auditory outcomes, SIN recognition was the most commonly assessed measure (19 studies), including 4 on-task and 15 off-task evaluations, with 6 studies reporting maintenance effects. Short-term SIN improvements were significant in 15 studies. Out of 6 follow-up studies, 4 [19,31,34,38] showed significant maintained benefits, while 2 [32,42,43] did not.

Pure-tone average (PTA) was assessed as an off-task outcome in 6 studies. Only 1 study [20] reported a significant short-term improvement (*F*_1,42_=3.12; *P*=.02; adjusted *P*=.06); the others showed no significant changes, and only 1 study reported a nonsignificant maintenance effect [38]. Insufficient data precluded meta-analysis.

Five studies evaluated self-reported hearing function outcomes. Three studies used the Speech, Spatial, and Qualities of Hearing Scale (SSQ); 2 studies found [32,38] no significant effects, and one found a significant effect [39]. Another 2 studies reported significant short-term improvements in subjective hearing difficulty (Hearing Handicap Inventory and Communication Scale for Older Adults for the Elderly/Adults) [39,45].

Auditory stream segregation task was evaluated in 2 studies [30,34], both reporting significant short-term improvements; only 1 study that included follow-up data confirmed the maintenance of this effect [34]. There were sufficient data to conduct a meta-analysis for this indicator.

#### Cognitive-Related Outcomes

Attention was evaluated in 9 studies. Six studies reported significant short-term improvements, whereas 3 found no significant effects. Three studies assessed the maintenance of treatment effects, with 2 [32,38] reporting sustained benefits and 1 [18] reporting no significant maintenance effect.

Short-term auditory memory was examined in 10 studies, with 6 reporting significant improvements; maintenance effects were mixed, with 2 studies showing retained benefits [19,38], and 3 studies showing no significant maintenance effect [18,32,42,43]. Four studies assessed episodic/verbal memory [18,20,31,38], mostly with nonsignificant short-term and maintenance effects.

Working memory outcomes showed domain-specific patterns. For auditory working memory, 3 of the 5 studies reported significant short-term improvements, whereas 2 [18,38] found no significant effects. Three studies assessed maintenance effects, with 2 [18,38] demonstrating sustained benefits and 1 [19] finding no significant maintenance effect. Similarly, for visual working memory, 3 of the 5 studies reported significant short-term improvements, whereas 2 [17,18] found no significant effects. Two studies [18,32] assessed maintenance, and 1 [32] reported a significant sustained benefit.

Processing speed was assessed in 4 studies: 3 reported significant short-term improvements [15,33,42,43], 1 did not [31]; 1 study found no sustained maintenance effects [31].

Global cognition was assessed in 3 studies, all of which used researcher-developed ACT training systems. One study reported a significant short-term improvement in MoCA scores [19], whereas the other 2 studies found no significant effects, with one assessing the RBANS (Repeatable Battery for Assessment of Neuropsychological Status) total score [18] and the other assessing MoCA scores [45]. One study assessed maintenance effects at follow-up but found no significant maintenance effects [18].

### Meta-Analysis Results

#### Hearing-Related Outcomes

The forest plots for the meta-analyses of auditory-related outcomes are presented in Figure 3. Fourteen studies assessing SIN perception were included in the meta-analysis. A random-effects model demonstrated a significant pooled effect (MD=−1.23, 95% CI −2.35 to −0.10; *P*=.03), with substantial heterogeneity (*I*²=87.2%). Egger regression test showed no significant evidence of funnel plot asymmetry (*t*=−1.33; *P*=.21; Figure S1 in Multimedia Appendix 1).

We also conducted a subgroup analysis to explore whether HA use contributed to the heterogeneity in the effects of ACT on SIN performance. Studies were stratified according to HA use into 2 subgroups: HA use (Yes) and no HA use (No). Among participants who did not use HAs, ACT was associated with a significant improvement in SIN performance (SMD=−0.32, 95% CI −0.52 to −0.11; *P*=.006), with low heterogeneity across studies (*I*²=9.3%). In contrast, among HA users, the pooled effect was not statistically significant (SMD=−0.87, 95% CI −1.76 to 0.02; *P*=.06), with substantial heterogeneity (*I*²=92.0%). The test for subgroup differences was not statistically significant (*χ*²_1_=1.38; *P*=.24), indicating no evidence of a statistically significant difference in the effects of ACT between participants who used HAs and those who did not (Figure S2 in Multimedia Appendix 1).

Two studies assessed PTA, with no significant pooled effect under a fixed-effect model (MD=−1.25, 95% CI −3.78 to 1.27; *P*=.33; *I*²=0%). Three studies assessed self-reported hearing outcomes, which also showed no significant pooled effect (MD=0.05, 95% CI −0.61 to 0.72; *P*=.87; *I*²=0%).

**Figure 3.**
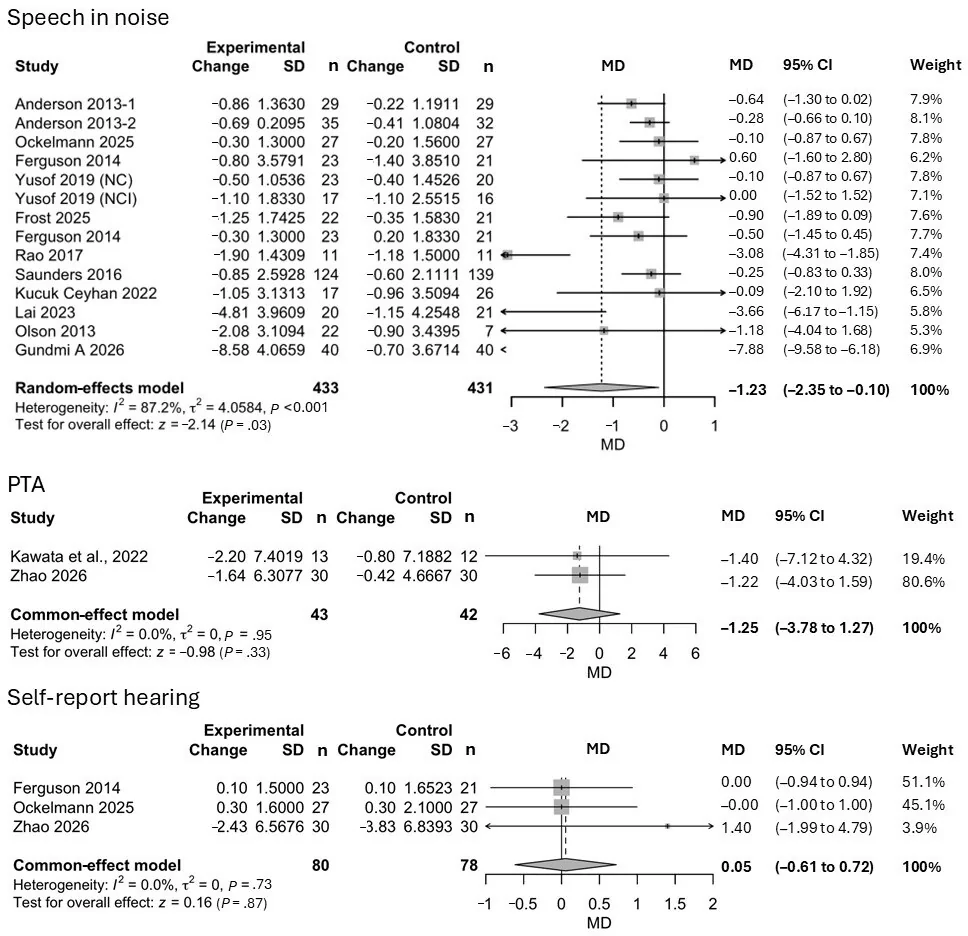
Forest plots for the meta-analyses of auditory-cognitive training on hearing-related outcomes [14,15,19,20,31-33,37-40,42,45]. MD: mean difference; PTA: pure-tone average.

#### Cognitive-Related Outcomes

The forest plots for the meta-analyses of cognitive-related outcomes are presented in Figure 4. Three studies assessing global cognition using the MoCA were included, with a significant pooled effect favoring the intervention group (MD=3.27, 95% CI 0.27-6.26; *P*=.03; *I*²=89.7%). Seven studies assessing attention showed a significant overall effect (SMD=0.50, 95% CI 0.23-0.77; *P*<.001; *I*²=29.5%). Eight studies assessing memory also demonstrated a significant pooled effect (SMD=1.08, 95% CI 0.25-1.92; *P*=.01), with substantial heterogeneity (*I*²=94.0%). Subgroup analysis showed positive pooled effects for both short-term memory (SMD=1.07, 95% CI −0.27 to 2.41; *I*²=96.0%) and delayed memory (SMD=0.96, 95% CI −0.01 to 1.93; *I*²=84.4%), but neither subgroup reached statistical significance. There was no significant difference between the 2 subgroups (*P*=.90). Leave-one-out sensitivity analysis did not identify any single study as a major contributor to the observed heterogeneity. In contrast, four studies assessing working memory showed no significant overall effect (SMD=0.15, 95% CI −0.32 to 0.63; *P*=.53; *I*²=53.4%). Two studies assessing processing speed also showed no significant pooled effect (SMD=1.79, 95% CI −1.66 to 5.25; *P*=.31; *I*²=98.0%).

**Figure 4.**
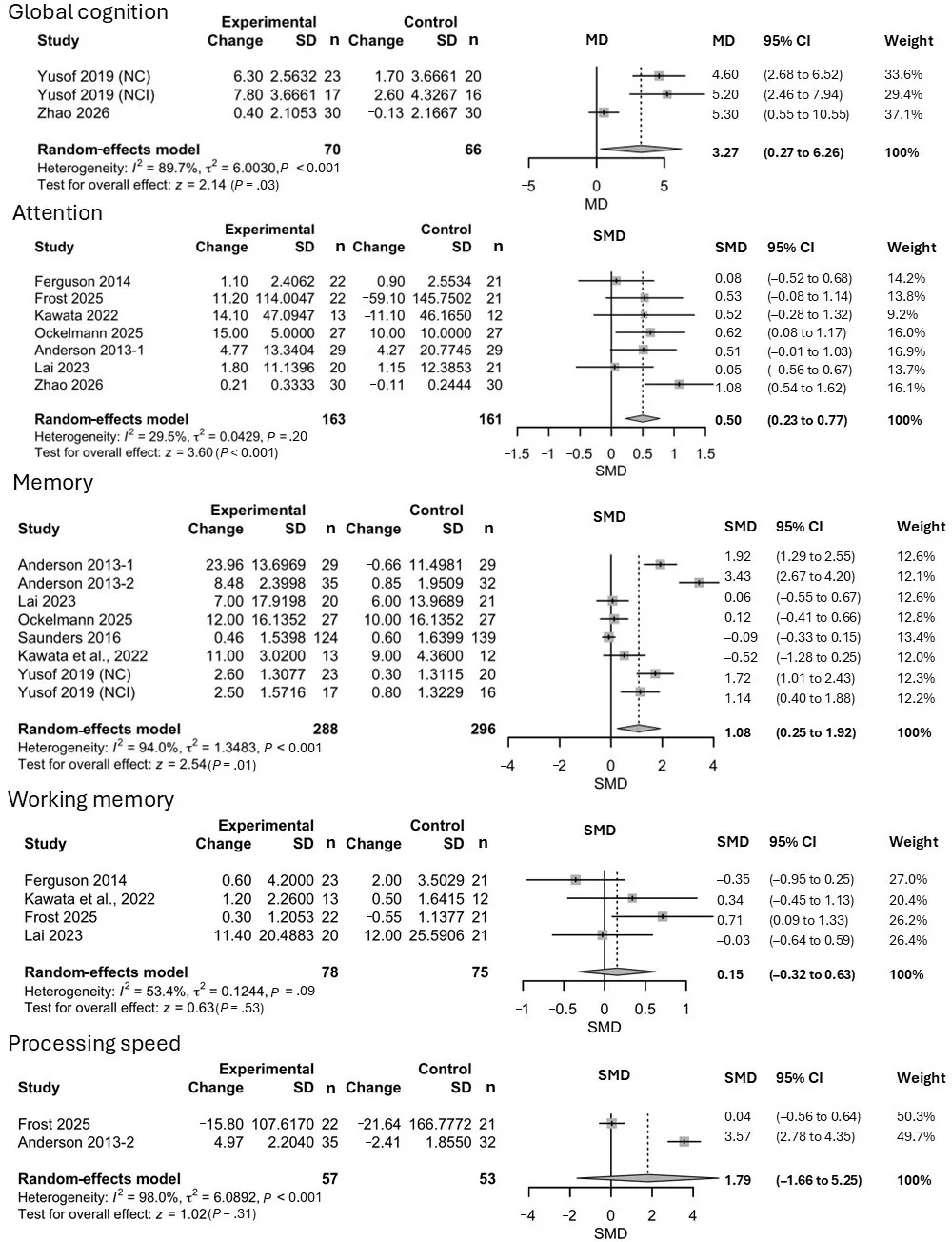
Forest plots for meta-analyses of auditory-cognitive training on cognitive-related outcomes [14,15,19,20,31,32,37,38,42,45]. MD: mean difference; SMD: standardized mean difference.

#### Summary of Findings

Table 3 summarizes the certainty of evidence for the main outcomes assessed using the GRADE (Grading of Recommendations, Assessment, Development, and Evaluation) approach [46]. The certainty of evidence ranged from very low to moderate. Attention was rated as moderate certainty, whereas SIN performance, memory, and self-reported hearing were rated as low certainty. Global cognition, PTA, and processing speed were rated as very low certainty. The certainty of evidence was mainly downgraded because of risk of bias, substantial heterogeneity, and imprecision due to limited sample sizes and wide CIs.

**Table 3.** Summary of meta-analysis findings.

Outcomes	Studies (n)	Participants (n)		Effect estimate (95% CI)	Certainty of evidence^b^
		ACT^a^ group	Control group		
Speech in noise	14	433	431	MD^c^: –1.23 (–2.35 to –0.10)	⊕⊕○○
Pure-tone average (dB)	2	43	42	MD: −1.25 (−3.78 to 1.27)	⊕○○○
Self-reported hearing	3	80	78	MD: 0.05 (−0.61 to 0.72)	⊕⊕○○
Global cognition	3	70	66	MD: 3.27 (0.27 to 6.26)	⊕○○○
Attention	7	163	161	SMD^d^: 0.50 (0.23 to 0.77)	⊕⊕⊕○
Memory	8	288	296	SMD: 1.08 (0.25 to 1.92)	⊕⊕○○
Working memory	4	78	75	SMD: 0.15 (−0.32 to 0.63)	⊕○○○
Processing speed	2	57	53	SMD: 1.79 (−1.66 to 5.25)	⊕○○○

a ACT: auditory-cognitive training.

b GRADE certainty of evidence: ⊕⊕⊕○, moderate certainty; ⊕⊕○○, low certainty; and ⊕○○○, very low certainty.

c MD: mean difference.

d SMD: standardized mean difference.

## Discussion

### Principal Findings

This mixed synthesis is the first to summarize ACT in older adults. A total of 21 studies (24 articles) involving 1300 older adults from 10 countries (the United States, United Kingdom, Switzerland, Japan, Malaysia, Germany, Turkey, Singapore, India, and China) were included. This review comprehensively summarized key features of existing ACT interventions, including participants’ hearing characteristics, delivery modes, training content, frequency and dosage, as well as outcome-specific effects. Considerable heterogeneity was observed in target populations’ hearing status, and ACT was delivered via diverse modalities including computer-based, mobile app, VR, and face-to-face training. Training content fell into 3 categories: auditory training, auditory-cognitive integrated training, and cognitive training, with a median total training dosage of 10 (IQR 1.5-60) hours. Narrative evidence indicated consistent short-term benefits of ACT for SIN recognition, attention, and auditory memory, with some benefits maintained at follow-up. Meta-analyses further supported significant beneficial effects of ACT on SIN recognition, attention, overall memory, and global cognition, although substantial heterogeneity was observed for SIN, memory, and global cognition. In contrast, pooled effects were not significant for working memory, processing speed, self-reported hearing outcomes, or PTA, while evidence for auditory stream segregation was limited but consistently positive.

Current evidence shows that ACT has been applied to older adults across a range of hearing levels for both preventive and rehabilitative purposes. It has been used not only in those with mild-to-moderate HL to improve speech perception but also in older adults with normal audiometric thresholds who report subjective hearing difficulties to reduce listening effort and cognitive load. ACT has also been used as an adjunct to audiological rehabilitation before or after HA fitting, suggesting potential value in boosting auditory and cognitive outcomes. Most existing ACT studies have been conducted in older adults with normal cognition or mild cognitive impairment. Notably, Ferguson et al [32] reported that cognitively normal participants achieved greater ACT-related improvements in SIN perception, gap detection, and pitch pattern sequencing than did those with mild cognitive impairment. However, this between-group difference should be interpreted cautiously, as it may partly reflect regression to the mean or baseline differences rather than differential responsiveness to ACT. Thus, the extent to which baseline cognitive status moderates ACT effectiveness remains uncertain. For those with existing cognitive impairment, higher training dosage and broader multidomain interventions (eg, physical activity and psychosocial approaches) may warrant consideration [47], highlighting the need for tailored protocols based on baseline cognitive function.

To date, no standardized ACT protocols exist, with substantial variability in delivery modalities, content, and dosage. Computer-based and mobile app–based interventions are more accessible and flexible than face-to-face or VR training, supporting higher adherence through shorter, more frequent sessions, with a median total dosage of 10 hours. Integrated ACT appears superior to unimodal auditory or cognitive training alone. For example, Faust et al [18] found that separate auditory or visual cognitive training improved modality-specific functions, but simple coadministration did not produce synergistic benefits, emphasizing the importance of genuine integration (eg, combining speech perception with memory or attention demands) rather than parallel task delivery. Common integrated designs pair auditory stimuli with demands on short-term/working memory, attention, processing speed, or executive function. All included ACT interventions used adaptive difficulty. Although only one study [20] directly compared adaptive and fixed difficulty, adaptive protocols may better accommodate individual learning rates and heterogeneous baseline abilities in older adults, thereby improving engagement and training outcomes.

Regarding intervention effects, the most consistent improvement was observed in SIN recognition, with benefits maintained for 1 to 2 months postintervention [34,38], and short-term effects supported by a meta-analysis. A potential mechanism is that ACT may reduce excessive subcortical encoding of speech envelopes, strengthen top-down attentional control, and enhance functional connectivity between the prefrontal and auditory cortices, thereby improving temporal processing and SIN perception [14,15]. Nevertheless, the pooled estimate showed substantial heterogeneity (*I*²=87.2%), which may partly reflect differences in SIN assessment paradigms, including HINT (Hearing-in-Noise Test), WIN (Words-in-Noise test), QuickSIN (Quick Speech-in-Noise), speech-in-babble, and competing-speaker tasks, as well as differences in participants’ baseline hearing status and HA use. The pooled effect indicated a significant reduction in signal-to-noise ratio/speech reception threshold of approximately 1.23 dB following ACT (MD=−1.23, 95% CI −2.35 to −0.10 dB), suggesting a clinically meaningful improvement in speech recognition under noisy conditions [48]. However, the high between-study heterogeneity indicates that the magnitude of benefit varied considerably across studies. Subgroup analysis by HA use provided some additional insight into this heterogeneity. Among participants who did not use HAs, the pooled effect was significant and showed low heterogeneity (*I*²=9.3%), whereas the effect among HA users was not statistically significant and was accompanied by substantial heterogeneity (*I*²=92.0%). Although the test for subgroup differences was not statistically significant (*P*=.24), the marked difference in within-subgroup heterogeneity suggests that HA use may be one potential contributor to the variability in treatment effects across studies. This modest improvement may also have limited implications for real-world hearing function, consistent with the nonsignificant pooled effect for self-reported hearing outcomes (SSQ). By contrast, ACT showed minimal effects on pure-tone thresholds, consistent with the limited reversibility of age-related cochlear degeneration [49]. This may be because ACT primarily targets central auditory and cognitive processing rather than peripheral hearing sensitivity.

For cognitive outcomes, the meta-analysis showed significant pooled effects for global cognition, attention, and memory, whereas the effects for working memory and processing speed were not statistically significant. Among these outcomes, the evidence for attention was the most consistent, with a significant effect (SMD=0.50, 95% CI 0.23-0.77) and low heterogeneity (*I*²=29.5%); moreover, the certainty of evidence was rated as moderate. This finding suggests that ACT may have a relatively robust beneficial effect on attention, potentially because the integration of auditory processing with cognitive control, selective attention, and task monitoring repeatedly engages overlapping auditory-cognitive processes. Such integrated training may strengthen the ability to allocate and maintain attention to relevant auditory information, particularly under cognitively demanding listening conditions. The pooled effect for memory was statistically significant but accompanied by substantial heterogeneity, and neither subgroup analysis by memory domain nor leave-one-out sensitivity analysis identified a major contributor to this heterogeneity. Therefore, the memory findings should be interpreted cautiously, given the substantial between-study variability and uncertainty regarding the magnitude and generalizability of the effect. For global cognition, the significant pooled effect should also be interpreted cautiously because it was based on a small number of studies and was accompanied by substantial heterogeneity. Similarly, the nonsignificant findings for working memory and processing speed provide insufficient evidence to determine whether ACT produces consistent benefits in these domains, particularly given the limited number of studies and variability in outcome measures and intervention characteristics. Overall, the current evidence suggests that the cognitive effects of ACT may vary across domains, with relatively stronger and more consistent evidence for attention than for broader or more specific cognitive functions. Evidence regarding the longer-term maintenance of cognitive benefits remains limited, particularly because of the scarcity of follow-up assessments, heterogeneous measures, and small sample sizes. Further well-designed trials using standardized cognitive outcomes, larger samples, and longer follow-up periods are needed to clarify the magnitude, generalizability, and durability of these cognitive effects.

We note several limitations when interpreting both the included evidence and our synthesis. At the evidence level, most studies used passive or waitlist control groups, making it difficult to distinguish the specific effects of ACT from those of single-domain auditory or cognitive training. Commercial training platforms (eg, CogniFit) may offer limited customization for SIN tasks and limited crosslinguistic adaptability, which may reduce ecological validity and generalizability. Few studies considered stimulus delivery modes (eg, headphones vs loudspeakers), which may affect listening comfort and effort in older adults with HL. In addition, most studies enrolled cognitively normal participants; only one study directly compared responses between cognitively normal and cognitively impaired groups. Thus, the efficacy of ACT for older adults with cognitive impairment remains uncertain. Few studies used patient-reported real-life listening outcomes, such as subjective hearing difficulty and daily listening function, as primary end points. Given the importance of subjective hearing experience for communication confidence, social participation, mental health, and quality of life [50], future studies should incorporate more patient-reported measures and longer follow-up periods to assess the persistence and real-life relevance of training benefits.

Our review also has its own limitations. First, we restricted inclusion to English-language publications, which may have introduced language bias and led to the omission of relevant evidence published in other languages. Although the included evidence covered several non–English-speaking countries, including Japan, Germany, and Malaysia, relevant studies published in local languages may have been overlooked. This limitation may reduce the completeness and crosslinguistic representativeness of the evidence base and should be considered when interpreting the generalizability of the findings. Second, although meta-analyses were performed for key auditory and cognitive outcomes, pooled effects should be interpreted cautiously because of substantial heterogeneity and the small number of studies contributing to some outcomes. Variability in construct definitions, training dosage, delivery modes, and assessment time points may have reduced comparability across studies. Third, limited follow-up data prevented quantitative synthesis of maintenance effects across domains. Despite these limitations, this synthesis integrated narrative and meta-analytic evidence to characterize outcome patterns, quantify short-term effects, and identify key research gaps, thereby providing a basis for the standardization, refinement, and mechanistic investigation of ACT interventions for older adults.

### Conclusions

This review systematically synthesizes the current evidence on ACT for older adults through a combined scoping review and meta-analytic approach. This review indicates that ACT may improve selected auditory and cognitive outcomes in older adults, particularly SIN perception, attention, memory, and global cognition. However, the certainty of the evidence was low for most outcomes, with substantial heterogeneity observed for several pooled effects. Evidence for changes in hearing thresholds, self-reported hearing function, working memory, and processing speed remains limited or inconclusive, and evidence for long-term maintenance is insufficient. These findings support the potential of ACT as a complementary approach to promote auditory-cognitive function in older adults, but do not yet establish its clinical effectiveness. Future trials should use standardized and adaptable ACT protocols, include patient-reported real-life listening outcomes, recruit more diverse samples with varied cognitive and hearing profiles, and incorporate longer follow-up periods. Establishing evidence-based protocols and clinically meaningful outcome benchmarks will be important for determining how ACT can be integrated into individualized hearing and cognitive health interventions.

## Supplementary material

10.2196/101632 Multimedia Appendix 1 Database searching strategies and results; intervention and control characteristics of included studies (Table S1); detailed outcomes of included studies (Table S2); funnel plot for SIN (Figure S1); and subgroup analysis of ACT effects on SIN performance by hearing aid use (Figure S2).

10.2196/101632 Multimedia Appendix 2 Quality assessment results (RoB-2 for RCT and JBI critical appraisal qualitative Evidence Syntheses).

10.2196/101632 Multimedia Appendix 3 The detailed narrative summary of each outcome.

10.2196/101632 Checklist 1 PRISMA checklist.

10.2196/101632 Checklist 2 PRISMA-ScR checklist.

## Abbreviations

ACT: auditory-cognitive training
APEX: Audiological Platform for Experimental Research
ELU: Ease of Language Understanding
GRADE: Grading of Recommendations, Assessment, Development, and Evaluation
HA: hearing aid
HELIX: Hearing and Listening Experience
HINT: Hearing-in-Noise Test
HL: hearing loss
JBI: Joanna Briggs Institute
MD: mean difference
MMSE: Mini-Mental State Examination
MoCA: Montreal Cognitive Assessment
OSF: Open Science Framework
PRISMA: Preferred Reporting Items for Systematic Reviews and Meta-Analyses
PRISMA-ScR: Preferred Reporting Items for Systematic Reviews and Meta-Analyses extension for Scoping Reviews
PTA: pure-tone average
QuickSIN: Quick Speech-in-Noise
RBANS: Repeatable Battery for Assessment of Neuropsychological Status
RCT: randomized controlled trial
RoB 2: revised Cochrane risk-of-bias tool
SIN: speech-in-noise
SMD: standardized mean difference
SSQ: Speech, Spatial, and Qualities of Hearing Scale
VR: virtual reality
WIN: Words-in-Noise test
