# Supplementary material for: Methods and Effectiveness of Auditory-Cognitive Training for Older Adults: Scoping Review and Meta-Analysis
Source: JMIR Rehabil Assist Technol. 2026 Nov 9;13:e101632. doi: 10.2196/101632 (PMC13747269; doi:10.2196/101632)
Supplement: Supplementary file 1 [file rehab-v13-e101632-s002.pdf]

RCT-ROB2

Study ID	Domain 1. Randomization process				Domain 2. Deviations from intended interventions							
	1.1	1.2	1.3	1.0 Assessor's J	2.1	2.2	2.3	2.4	2.5	2.6	2.7	2.0 Assessor's .
Anderson 20	PY	NI	N	Some concerns	Y	NI	PN	NA	NA	Y	NA	Low
Anderson 20	PY	NI	N	Some concerns	N	PN	NA	NA	NA	Y	NA	Low
Abrams 2015	PY	NI	N	Some concerns	Y	NI	Y	Y	Y	Y	NA	Some concerns
Faust 2020	PY	NI	N	Some concerns	Y	PN	PN	NA	NA	Y	NA	Low
Ferguson 20	Y	NI	N	Some concerns	Y	PN	PN	NA	NA	Y	NA	Low
Fisher 2025	PN	NI	PN	High	Y	PN	PN	NA	NA	Y	NA	Low
Frei 2025	PY	NI	PN	Some concerns	Y	PY	PN	NA	NA	Y	NA	Low
Frost 2025	Y	NI	N	Some concerns	PN	PN	NA	NA	NA	Y	NA	Low
Gundmi 2026	Y	Y	N	Low	Y	Y	PN	NA	NA	Y	NA	Low
Sweet&Hend	PY	NI	PN	Some concerns	Y	PN	NI	NA	NA	Y	NA	Some concerns
Kawata 2022	Y	Y	N	Low	Y	PY	PN	NA	NA	Y	NA	Low
Ockelmann 2	PY	NI	N	Some concerns	Y	PN	PN	NA	NA	Y	NA	Low
Rishiq 2016	PY	NI	N	Some concerns	Y	PN	PN	NA	NA	Y	NA	Low
Saunders 20	Y	Y	N	Low	Y	Y	PN	NA	NA	Y	NA	Low
Whitton 2017	Y	Y	N	Low	N	PN	NA	NA	NA	Y	NA	Low
Zhao 2026	Y	Y	N	Low	Y	PN	PN	NA	NA	Y	NA	Low

Study ID	Domain 3. Mising outcome data					Domain 4. Measurement of the ou						Domain 5. Selection of the re				Domain 6. Overall B
	3.1	3.2	3.3	3.4	3.0 Assessor's judgement	4.1	4.2	4.3	4.4	4.5	4.0 As	5.1	5.2	5.3	5.0 Assessc	Assessor's overall Judgement
Anderson 20	Y	NA	NA	NA	Low	N	N	NI	PN	NA	Low	N	PN	PN	Some conce	Some concerns
Anderson 20	Y	NA	NA	NA	Low	N	N	N	NA	NA	Low	NI	PN	PN	Some conce	Some concerns
Abrams 2015	Y	NA	NA	NA	Low	N	N	NI	PN	NA	Low	NI	PN	PN	Some conce	Some concerns
Faust 2020	NI	PN	NI	PN	Some concerns	N	N	N	NA	NA	Low	N	PN	PN	Some conce	Some concerns
Ferguson 20	Y	NA	NA	NA	Low	N	N	NI	PN	NA	Low	NI	PN	PN	Some conce	Some concerns
Fisher 2025	Y	NA	NA	NA	Low	N	N	NI	PN	NA	Low	NI	PN	PN	Some conce	High
Frei 2025	Y	NA	NA	NA	Low	N	N	Y	PN	NA	Low	NI	PN	PN	Some conce	Some concerns
Frost 2025	PY	NA	NA	NA	Low	N	N	Y	PN	NA	Low	NI	PN	PN	Some conce	Some concerns
Gundmi 2026	Y	NA	NA	NA	Low	N	N	N	NA	NA	Low	N	PN	PN	Some conce	Some concerns
Sweet&Hend	PN	N	N	NA	Low	N	N	NI	PN	NA	Low	NI	PN	PN	Some conce	Some concerns
Kawata 2022	Y	NA	NA	NA	Low	N	N	NI	PN	NA	Low	Y	PN	PN	Low	Low
Ockelmann 2	PY	NA	NA	NA	Low	N	N	NI	PN	NA	Low	NI	PN	PN	Some conce	Some concerns
Rishiq 2016	Y	NA	NA	NA	Low	N	N	NI	PN	NA	Low	N	NI	PN	Some conce	Some concerns
Saunders 20	PY	NA	NA	NA	Low	N	N	Y	PN	NA	Low	NI	PN	PN	Some conce	Some concerns
Whitton 2017	PY	NA	NA	NA	Low	N	N	N	NA	NA	Low	Y	PN	PN	Low	Low
Zhao 2026	Y	NA	NA	NA	Low	N	N	N	NA	NA	Low	Y	PN	PN	Low	Low

Note: Y: Yes, PY: Probably Yse; N: No, PN: Probably No; NI: No Information; NA: Not available

Summary

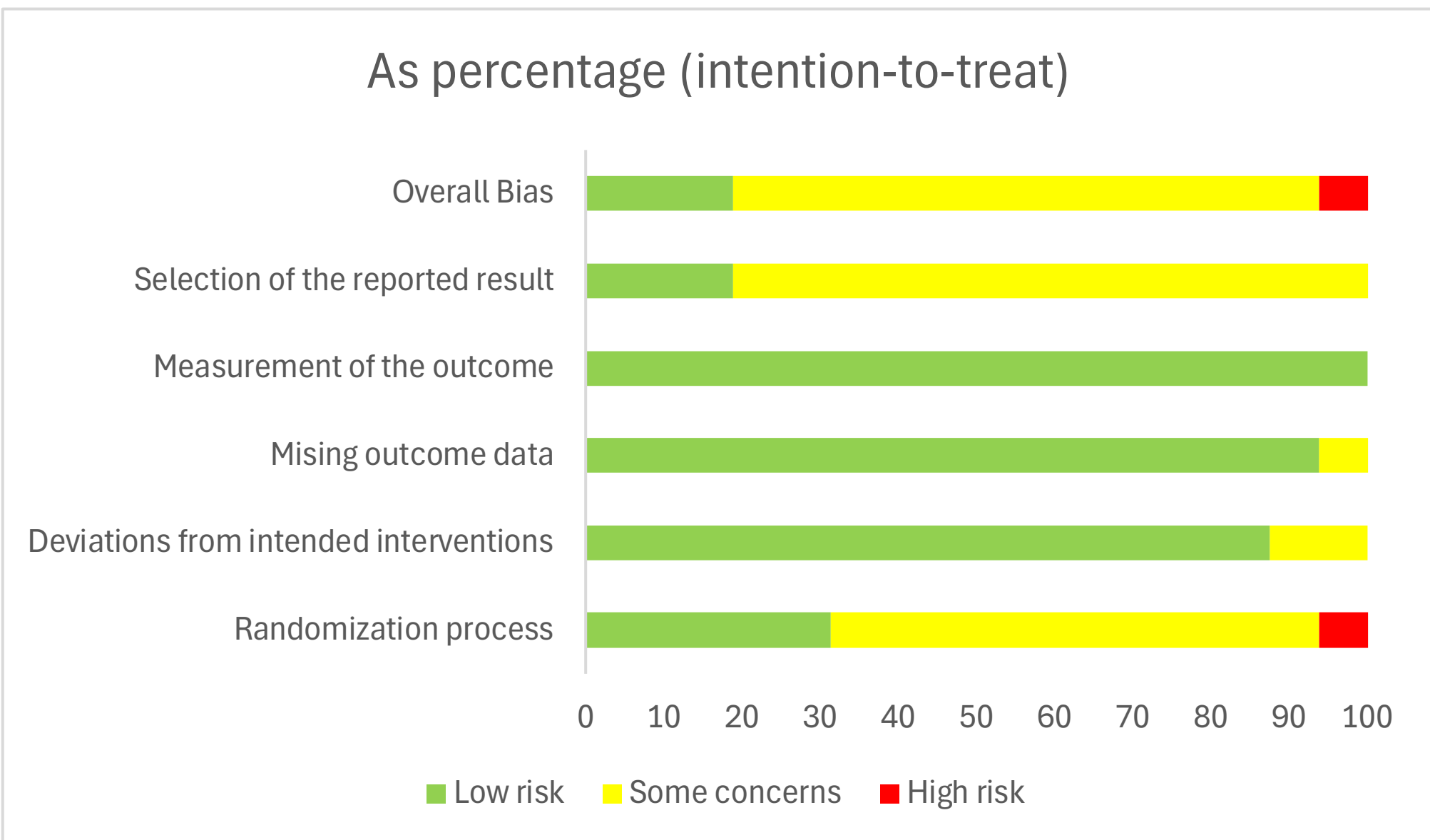

Study ID	D1	D2	D3	D4	D5	Overall
Anderson 2013b	!	+	+	+	!	!
Abrams 2015	!	!	+	+	!	!
Faust 2020	!	+	!	+	!	!
Ferguson 2014	!	+	+	+	!	!
Fisher 2025	-	+	+	+	!	-
Frei 2025	!	+	+	+	!	!
Frost 2025	!	+	+	+	!	!
Gundmi 2026	+	+	+	+	!	!
Sweet&Henderson 2006	!	!	+	+	!	!
Kawata 2022	+	+	+	+	+	+
Ockelmann 2025	!	+	+	+	!	!
Rishiq 2016	!	+	+	+	!	!
Saunders 2016	+	+	+	+	!	!
Whitton 2017	+	+	+	+	+	+
Zhao 2026	+	+	+	+	+	+
Anderson 2013a	!	+	+	+	!	!

+ Low risk
! Some concerns
- High risk

D1 Randomisation process
D2 Deviations from the intended interventions
D3 Missing outcome data
D4 Measurement of the outcome
D5 Selection of the reported result

JBI Critical apprisal of Q-Es

Study ID	Bias related to temporal precedence	Bias related to selection and allocation	Bias related to confounding factors	Bias related to administration of intervention/exposure				Bias related to participant retention	Statistical Conclusion Validity
	1	2	3	4	5	6	7	8	9
Yusof 2019	Y	Y	Y	Y	Y	Y	Y	Y	Y
Kucuk 2022	Y	Y	Y	UC	N	Y	Y	Y	Y
Lai 2023	Y	Y	Y	Y	N	Y	Y	Y	Y
Olson 2013	Y	Y	Y	UC	Y	Y	Y	Y	Y
Rao 2017	Y	Y	Y	Y	Y	Y	Y	Y	Y

Note: Y, Yes; N, No; UC, Unclear
1. Is it clear in the study what is the “cause” and what is the “effect” (i.e. there is no confusion about which variable comes first)?
2.Was there a control group?
3.Were participants included in any comparisons similar?
4.Were the participants included in any comparisons receiving similar treatment/care, other than the exposure or intervention of interest?
5.Were there multiple measurements of the outcome, both pre and post the intervention/exposure?
6.Were the outcomes of participants included in any comparisons measured in the same way?
7.Were outcomes measured in a reliable way?
8.Was follow-up complete and if not, were differences between groups in terms of their follow-up adequately described and analyzed?
9.Was appropriate statistical analysis used?

References

1. Anderson, S., et al., Training changes processing of speech cues in older adults with hearing loss. Front Syst Neurosci, 2013. 7: p. 97.
2. Anderson, S., et al., Reversal of age-related neural timing delays with training. Proceedings of the National Academy of Sciences of the United States of America, 2013. 110(11): p. 4357-4362.
3. Abrams, H.B., K. Bock, and R.L. Irey, Can a Remotely Delivered Auditory Training Program Improve Speech-in-Noise Understanding? Am J Audiol, 2015. 24(3): p. 333-7.
4. Faust, M.E., et al., Exploring the Specificity, Synergy, and Durability of Auditory and Visual Computer Gameplay Transfer Effects in Healthy Older Adults. The journals of gerontology. Series B, Psychological sciences and social sciences, 2020. 75(6): p. 1170-1180.
5. Ferguson, M.A., et al., Benefits of phoneme discrimination training in a randomized controlled trial of 50- to 74-year-olds with mild hearing loss. Ear Hear, 2014. 35(4): p. e110-21.
6. Fisher, C., et al., The Impacts of Two Adaptive Auditory-Cognitive Training Paradigms on Listening to Competing Talkers. Seminars in Hearing, 2025. 46(2): p. 141-160.
7. Fisher, C., et al., Neural and Behavioral Changes in Older Adults from Auditory-Cognitive Training. Annual International Conference of the IEEE Engineering in Medicine and Biology Society. IEEE Engineering in Medicine and Biology Society. Annual International Conference, 2025. 2025: p. 1-6.
8. Frei, V. and N. Giroud, Immersive auditory-cognitive training improves speech-in-noise perception in older adults with varying hearing and working memory. NPJ Sci Learn, 2025. 10(1): p. 12.
9. Frost, E., et al., HEaring and LIstening eXperience (HELIX): Evaluation of a co-designed serious game for auditory-cognitive training. Q J Exp Psychol (Hove), 2025: p. 17470218251324168.
10. Gundmi, A., et al., Efficacy of a structured auditory training module in older individuals with hearing loss: a randomized controlled

11. Henderson Sabes, J. and R.W. Sweetow, Variables predicting outcomes on listening and communication enhancement (LACE) training. Int J Audiol, 2007. 46(7): p. 374-83.
12. Sweetow, R.W. and J.H. Sabes, The need for and development of an adaptive Listening and Communication Enhancement (LACE) Program. J Am Acad Audiol, 2006. 17(8): p. 538-58.
13. Kawata, N.Y.S., et al., Auditory Cognitive Training Improves Brain Plasticity in Healthy Older Adults: Evidence From a Randomized Controlled Trial. Front Aging Neurosci, 2022. 14: p. 826672.
14. Ockelmann, J., et al., Personalized and gamified auditory-cognitive training improves naturalistic speech-in-noise comprehension in older adults with hearing loss. NPJ Sci Learn, 2025. 10(1): p. 80.
15. Rishiq, D., et al., Can a Commercially Available Auditory Training Program Improve Audiovisual Speech Performance? Am J Audiol, 2016. 25(3s): p. 308-312.
16. Saunders, G.H., et al., A Randomized Control Trial: Supplementing Hearing Aid Use with Listening and Communication Enhancement (LACE) Auditory Training. Ear Hear, 2016. 37(4): p. 381-96.
17. Smith, S.L., et al., Examination of Individual Differences in Outcomes From a Randomized Controlled Clinical Trial Comparing Formal and Informal Individual Auditory Training Programs. J Speech Lang Hear Res, 2016. 59(4): p. 876-86.
18. Whitton, J.P., et al., Audiomotor Perceptual Training Enhances Speech Intelligibility in Background Noise. Curr Biol, 2017. 27(21): p. 3237-3247.e6.
19. Zhao, I.Y., et al., Web-Based Gamified Auditory-Cognitive Dual-Task Training for Older Adults With Age-Related Hearing Loss: Pilot Randomized Controlled Trial. JMIR Aging, 2026. 9: p. e84083.
20. Yusof, Y., et al., Efficacy of a newly developed auditory–cognitive training system on speech recognition, central auditory processing and cognitive ability among older adults with normal cognition and with neurocognitive impairment. Geriatrics and

21. Kucuk Ceyhan, A., H.H. Dere, and B. Mujdeci, Evaluating the Effectiveness of a New Auditory Training Program on the Speech Recognition Skills and Auditory Event-Related Potentials in Elderly Hearing Aid Users. Audiol Neurootol, 2022. 27(5): p. 368-376.
22. Lai, C.Y., et al., Effects of Auditory Training in Older Adults. J Speech Lang Hear Res, 2023. 66(10): p. 4137-4149.
23. Olson, A.D., J.E. Preminger, and J.B. Shinn, The effect of LACE DVD training in new and experienced hearing aid users. J Am Acad Audiol, 2013. 24(3): p. 214-30.
24. Rao, A., et al., Neural Correlates of Selective Attention With Hearing Aid Use Followed by ReadMyQuips Auditory Training Program. Ear Hear, 2017. 38(1): p. 28-41.
