# Supplementary material for: Methods and Effectiveness of Auditory-Cognitive Training for Older Adults: Scoping Review and Meta-Analysis
Source: JMIR Rehabil Assist Technol. 2026 Nov 9;13:e101632. doi: 10.2196/101632 (PMC13747269; doi:10.2196/101632)
Supplement: Supplementary file 2 [file rehab-v13-e101632-s003.pdf]

## RCT-ROB2

| Study ID | Domain 1. Randomization process | | | | Domain 2. Deviations from intended interventions | | | | | | | | Domain 3. Mising outcome data | | | | | Domain 4. Measurement of the ou | | | | | | Domain 5. Selection of the re | | | | Domain 6. Overall B |
|---|---|---|---|---|---|---|---|---|---|---|---|---|---|---|---|---|---|---|---|---|---|---|---|---|---|---|---|---|
| | 1.1 | 1.2 | 1.3 | 1.0 Assessor's J | 2.1 | 2.2 | 2.3 | 2.4 | 2.5 | 2.6 | 2.7 | 2.0 Assessor's . | 3.1 | 3.2 | 3.3 | 3.4 | 3.0 Assessor's judgement | 4.1 | 4.2 | 4.3 | 4.4 | 4.5 | 4.0 As | 5.1 | 5.2 | 5.3 | 5.0 Assessc | Assessor's overall Judgement |
| Anderson 20 | PY | NI | N | Some concerns | Y | NI | PN | NA | NA | Y | NA | Low | Y | NA | NA | NA | Low | N | N | NI | PN | NA | Low | N | PN | PN | Some conce | Some concerns |
| Anderson 20 | PY | NI | N | Some concerns | N | PN | NA | NA | NA | Y | NA | Low | Y | NA | NA | NA | Low | N | N | N | NA | NA | Low | NI | PN | PN | Some conce | Some concerns |
| Abrams 2015 | PY | NI | N | Some concerns | Y | NI | Y | Y | Y | Y | NA | Some concerns | Y | NA | NA | NA | Low | N | N | NI | PN | NA | Low | NI | PN | PN | Some conce | Some concerns |
| Faust 2020 | PY | NI | N | Some concerns | Y | PN | PN | NA | NA | Y | NA | Low | NI | PN | NI | PN | Some concerns | N | N | N | NA | NA | Low | N | PN | PN | Some conce | Some concerns |
| Ferguson 20 | Y | NI | N | Some concerns | Y | PN | PN | NA | NA | Y | NA | Low | Y | NA | NA | NA | Low | N | N | NI | PN | NA | Low | NI | PN | PN | Some conce | Some concerns |
| Fisher 2025 | PN | NI | PN | High | Y | PN | PN | NA | NA | Y | NA | Low | Y | NA | NA | NA | Low | N | N | NI | PN | NA | Low | NI | PN | PN | Some conce | High |
| Frei 2025 | PY | NI | PN | Some concerns | Y | PY | PN | NA | NA | Y | NA | Low | Y | NA | NA | NA | Low | N | N | Y | PN | NA | Low | NI | PN | PN | Some conce | Some concerns |
| Frost 2025 | Y | NI | N | Some concerns | PN | PN | NA | NA | NA | Y | NA | Low | PY | NA | NA | NA | Low | N | N | Y | PN | NA | Low | NI | PN | PN | Some conce | Some concerns |
| Gundmi 2026 | Y | Y | N | Low | Y | Y | PN | NA | NA | Y | NA | Low | Y | NA | NA | NA | Low | N | N | N | NA | NA | Low | N | PN | PN | Some conce | Some concerns |
| Sweet&Hend | PY | NI | PN | Some concerns | Y | PN | NI | NA | NA | Y | NA | Some concerns | PN | N | N | NA | Low | N | N | NI | PN | NA | Low | NI | PN | PN | Some conce | Some concerns |
| Kawata 2022 | Y | Y | N | Low | Y | PY | PN | NA | NA | Y | NA | Low | Y | NA | NA | NA | Low | N | N | NI | PN | NA | Low | Y | PN | PN | Low | Low |
| Ockelmann 2 | PY | NI | N | Some concerns | Y | PN | PN | NA | NA | Y | NA | Low | PY | NA | NA | NA | Low | N | N | NI | PN | NA | Low | NI | PN | PN | Some conce | Some concerns |
| Rishiq 2016 | PY | NI | N | Some concerns | Y | PN | PN | NA | NA | Y | NA | Low | Y | NA | NA | NA | Low | N | N | NI | PN | NA | Low | N | NI | PN | Some conce | Some concerns |
| Saunders 20 | Y | Y | N | Low | Y | Y | PN | NA | NA | Y | NA | Low | PY | NA | NA | NA | Low | N | N | Y | PN | NA | Low | NI | PN | PN | Some conce | Some concerns |
| Whitton 2017 | Y | Y | N | Low | N | PN | NA | NA | NA | Y | NA | Low | PY | NA | NA | NA | Low | N | N | N | NA | NA | Low | Y | PN | PN | Low | Low |
| Zhao 2026 | Y | Y | N | Low | Y | PN | PN | NA | NA | Y | NA | Low | Y | NA | NA | NA | Low | N | N | N | NA | NA | Low | Y | PN | PN | Low | Low |

Note: Y: Yes, PY: Probably Yse; N: No, PN: Probably No; NI: No Information; NA: Not available

**Summary**

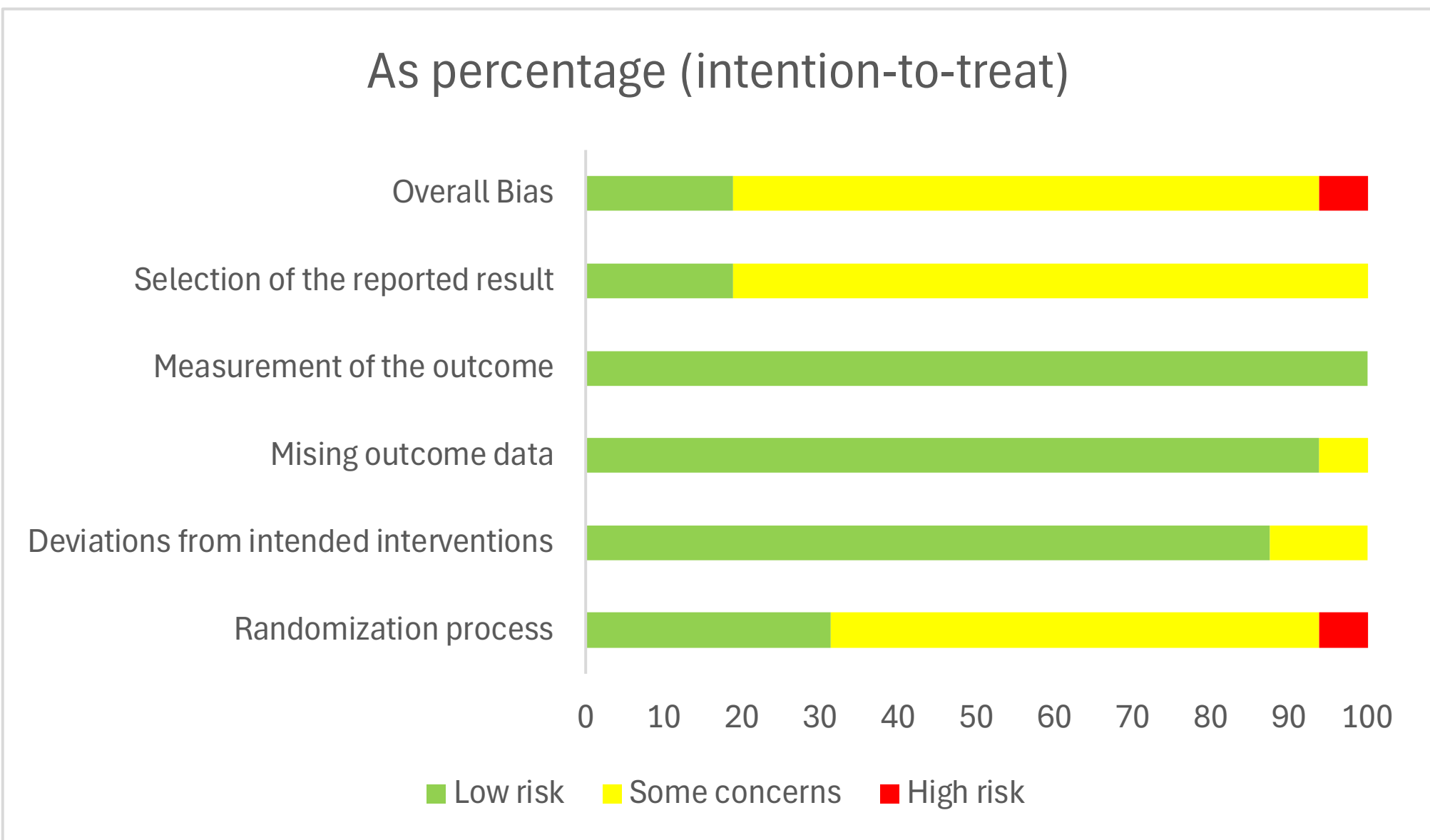


| Study ID | D1 | D2 | D3 | D4 | D5 | Overall |
|---|---|---|---|---|---|---|
| Anderson 2013b | ! | + | + | + | ! | ! |
| Abrams 2015 | ! | ! | + | + | ! | ! |
| Faust 2020 | ! | + | ! | + | ! | ! |
| Ferguson 2014 | ! | + | + | + | ! | ! |
| Fisher 2025 | - | + | + | + | ! | - |
| Frei 2025 | ! | + | + | + | ! | ! |
| Frost 2025 | ! | + | + | + | ! | ! |
| Gundmi 2026 | + | + | + | + | ! | ! |
| Sweet&Henderson 2006 | ! | ! | + | + | ! | ! |
| Kawata 2022 | + | + | + | + | + | + |
| Ockelmann 2025 | ! | + | + | + | ! | ! |
| Rishiq 2016 | ! | + | + | + | ! | ! |
| Saunders 2016 | + | + | + | + | ! | ! |
| Whitton 2017 | + | + | + | + | + | + |
| Zhao 2026 | + | + | + | + | + | + |
| Anderson 2013a | ! | + | + | + | ! | ! |

+ Low risk
! Some concerns
- High risk

D1 Randomisation process
D2 Deviations from the intended interventions
D3 Missing outcome data
D4 Measurement of the outcome
D5 Selection of the reported result

## JBI Critical apprisal of Q-Es

| Study ID | Bias related to temporal precedence | Bias related to selection and allocation | Bias related to confounding factors | | Bias related to administration of intervention/exposure | | | Bias related to participant retention | Statistical Conclusion Validity |
|---|---|---|---|---|---|---|---|---|---|
| | 1 | 2 | 3 | 4 | 5 | 6 | 7 | 8 | 9 |
| Yusof 2019 | Y | Y | Y | Y | Y | Y | Y | Y | Y |
| Kucuk 2022 | Y | Y | Y | UC | N | Y | Y | Y | Y |
| Lai 2023 | Y | Y | Y | Y | N | Y | Y | Y | Y |
| Olson 2013 | Y | Y | Y | UC | Y | Y | Y | Y | Y |
| Rao 2017 | Y | Y | Y | Y | Y | Y | Y | Y | Y |

Note: Y, Yes; N, No; UC, Unclear

1. Is it clear in the study what is the “cause” and what is the “effect” (i.e. there is no confusion about which variable comes first)?
2.Was there a control group?
3.Were participants included in any comparisons similar?
4.Were the participants included in any comparisons receiving similar treatment/care, other than the exposure or intervention of interest?
5.Were there multiple measurements of the outcome, both pre and post the intervention/exposure?
6.Were the outcomes of participants included in any comparisons measured in the same way?
7.Were outcomes measured in a reliable way?
8.Was follow-up complete and if not, were differences between groups in terms of their follow-up adequately described and analyzed?
9.Was appropriate statistical analysis used?

**References**


1. Anderson, S., et al., Training changes processing of speech cues in older adults with hearing loss. Front Syst Neurosci, 2013. 7: p. 97.
2. Anderson, S., et al., Reversal of age-related neural timing delays with training. Proceedings of the National Academy of Sciences of the United States of America, 2013. 110(11): p. 4357-4362.
3. Abrams, H.B., K. Bock, and R.L. Irey, Can a Remotely Delivered Auditory Training Program Improve Speech-in-Noise Understanding? Am J Audiol, 2015. 24(3): p. 333-7.
4. Faust, M.E., et al., Exploring the Specificity, Synergy, and Durability of Auditory and Visual Computer Gameplay Transfer Effects in Healthy Older Adults. The journals of gerontology. Series B, Psychological sciences and social sciences, 2020. 75(6): p. 1170-1180.
5. Ferguson, M.A., et al., Benefits of phoneme discrimination training in a randomized controlled trial of 50- to 74-year-olds with mild hearing loss. Ear Hear, 2014. 35(4): p. e110-21.
6. Fisher, C., et al., The Impacts of Two Adaptive Auditory-Cognitive Training Paradigms on Listening to Competing Talkers. Seminars in Hearing, 2025. 46(2): p. 141-160.
7. Fisher, C., et al., Neural and Behavioral Changes in Older Adults from Auditory-Cognitive Training. Annual International Conference of the IEEE Engineering in Medicine and Biology Society. IEEE Engineering in Medicine and Biology Society. Annual International Conference, 2025. 2025: p. 1-6.
8. Frei, V. and N. Giroud, Immersive auditory-cognitive training improves speech-in-noise perception in older adults with varying hearing and working memory. NPJ Sci Learn, 2025. 10(1): p. 12.
9. Frost, E., et al., HEaring and LIstening eXperience (HELIX): Evaluation of a co-designed serious game for auditory-cognitive training. Q J Exp Psychol (Hove), 2025: p. 17470218251324168.
10. Gundmi, A., et al., Efficacy of a structured auditory training module in older individuals with hearing loss: a randomized controlled

11. Henderson Sabes, J. and R.W. Sweetow, Variables predicting outcomes on listening and communication enhancement (LACE) training. Int J Audiol, 2007. 46(7): p. 374-83.
12. Sweetow, R.W. and J.H. Sabes, The need for and development of an adaptive Listening and Communication Enhancement (LACE) Program. J Am Acad Audiol, 2006. 17(8): p. 538-58.
13. Kawata, N.Y.S., et al., Auditory Cognitive Training Improves Brain Plasticity in Healthy Older Adults: Evidence From a Randomized Controlled Trial. Front Aging Neurosci, 2022. 14: p. 826672.
14. Ockelmann, J., et al., Personalized and gamified auditory-cognitive training improves naturalistic speech-in-noise comprehension in older adults with hearing loss. NPJ Sci Learn, 2025. 10(1): p. 80.
15. Rishiq, D., et al., Can a Commercially Available Auditory Training Program Improve Audiovisual Speech Performance? Am J Audiol, 2016. 25(3s): p. 308-312.
16. Saunders, G.H., et al., A Randomized Control Trial: Supplementing Hearing Aid Use with Listening and Communication Enhancement (LACE) Auditory Training. Ear Hear, 2016. 37(4): p. 381-96.
17. Smith, S.L., et al., Examination of Individual Differences in Outcomes From a Randomized Controlled Clinical Trial Comparing Formal and Informal Individual Auditory Training Programs. J Speech Lang Hear Res, 2016. 59(4): p. 876-86.
18. Whitton, J.P., et al., Audiomotor Perceptual Training Enhances Speech Intelligibility in Background Noise. Curr Biol, 2017. 27(21): p. 3237-3247.e6.
19. Zhao, I.Y., et al., Web-Based Gamified Auditory-Cognitive Dual-Task Training for Older Adults With Age-Related Hearing Loss: Pilot Randomized Controlled Trial. JMIR Aging, 2026. 9: p. e84083.
20. Yusof, Y., et al., Efficacy of a newly developed auditory–cognitive training system on speech recognition, central auditory processing and cognitive ability among older adults with normal cognition and with neurocognitive impairment. Geriatrics and
21. Kucuk Ceyhan, A., H.H. Dere, and B. Mujdeci, Evaluating the Effectiveness of a New Auditory Training Program on the Speech Recognition Skills and Auditory Event-Related Potentials in Elderly Hearing Aid Users. Audiol Neurootol, 2022. 27(5): p. 368-376.
22. Lai, C.Y., et al., Effects of Auditory Training in Older Adults. J Speech Lang Hear Res, 2023. 66(10): p. 4137-4149.
23. Olson, A.D., J.E. Preminger, and J.B. Shinn, The effect of LACE DVD training in new and experienced hearing aid users. J Am Acad Audiol, 2013. 24(3): p. 214-30.
24. Rao, A., et al., Neural Correlates of Selective Attention With Hearing Aid Use Followed by ReadMyQuips Auditory Training Program. Ear Hear, 2017. 38(1): p. 28-41.
