# Supplementary material for: Methods and Effectiveness of Auditory-Cognitive Training for Older Adults: Scoping Review and Meta-Analysis
Source: JMIR Rehabil Assist Technol. 2026 Nov 9;13:e101632. doi: 10.2196/101632 (PMC13747269; doi:10.2196/101632)
Supplement: Supplementary file 5 [file rehab-v13-e101632-s001.docx]

**Supplementary materials**

**Database searching strategies and results**

1. Pubmed: 466

("Hearing Loss"[Mesh] OR "Auditory Perception"[Mesh] OR "Auditory Perceptual Disorders"[Mesh]) AND "Aged"[Mesh]) AND ("auditory training" OR "auditory rehabilitation" OR “hearing rehabilitation” OR “Perceptual training”)

2. Embase: 62

(hearing* OR cochlear* OR 'deafness'/exp OR deafness OR 'hard of hearing'/exp OR 'hard of hearing') AND ('auditory cognitive training' OR (('perceptual learning'/exp OR 'perceptual learning' OR 'perceptual training'/exp OR 'perceptual training' OR 'auditory learning'/exp OR 'auditory learning' OR 'auditory training'/exp OR 'auditory training' OR 'music training'/exp OR 'music training' OR 'pitch'/exp OR 'pitch' OR 'timbre'/exp OR 'timbre' OR 'melody'/exp OR 'melody' OR 'melodic contour identification') AND ('cognitive training'/exp OR 'cognitive training' OR 'cognitive therapy'/exp OR 'cognitive therapy' OR 'cognitive rehabilitation'/exp OR 'cognitive rehabilitation' OR 'cognitive remediation'/exp OR 'cognitive remediation' OR 'cognitive intervention*' OR 'cognitive rehabilitation therapy')))

3. APA PsycNet: 106

Any Field: Auditory cognitive training AND (Any Field: Hearing* OR Any Field: Cochlear* OR Any Field: Deafness OR Any Field: "Hard of hearing") NOT Any Field: child OR Any Field: children OR Any Field: rat AND Any Field: "Adult*" OR Any Field: "Elder*" OR Any Field: "Older adult*"

4. Cochrane：12

(Hearing* OR Cochlear* OR Deafness OR "Hard of hearing") AND ( ("Auditory cognitive training") OR ( ("Perceptual learning" OR "Perceptual training" OR "Auditory learning" OR "Auditory training" OR "Music training" OR "Pitch" OR "Timbre" OR "Melody" OR "Melodic contour identification") AND ("Cognitive training" OR "Cognitive therapy" OR "Cognitive rehabilitation" OR "Cognitive remediation" OR "Cognitive intervention*" OR "Cognitive rehabilitation therapy") ) ) in Title Abstract Keyword

Table S1 Intervention and Control Characteristics of Included Studies

| **Study ID** | **Training Modality** | **ACT Domain** | **Control description** | **Training Duration & Frequency** | **Total training dosage (hour)** | **Follow up** | **Hearing-Related Measures** | **Cognition Related Measures** | **Other Measures** |
| --- | --- | --- | --- | --- | --- | --- | --- | --- | --- |
| Abrams 2015 [1] | Remote internet-based home computer audiovisual training | RMQ: audiovisual adaptive auditory training program delivered via games, puzzles and short videos. Specific training modules not reported. | Standard hearing aid fitting, routine maintenance counseling, periodic phone follow-up; No access to the RMQ auditory training platform | 4 weeks; 30 min/day; 5 days/week | prescribed: 7.5, actual: 3.84 ± 4.94 | Visit 1 (Baseline); Visit 2 (1 week post-baseline); Visit 3 (4 weeks post-baseline, immediately after training completion) | HINT, WIN | Not reported | None |
| Anderson 2013 [2] | Computer-based, home-based, gamified | Brain Fitness software (6 modules): A processing speed; syllable discrimination; sequence recognition; command execution; adaptive consonant-vowel transition perception | Watching educational DVDs + completing multiple-choice questions (no targeted A/C training) | 8 weeks; 1 hour/day; 5 days/week | 40 | pre-training and post-training | QuickSIN test | WJ_III: Auditory Numbers Reversed, and memory for Words; IVA: Sustained attention | FFR via EEG; speech envelope coding (F₀ADD, H₂ADD); TFS coding (H₃SUB-H₆SUB) |
| Anderson 2013 b [3] | Same as above | Same as above | Same as above | Same as above | 40 | pre-training and post-training | Same as above | WJ_III Auditory short-term memory and processing speed; MOCA | ABR to syllable; FFR peak latency and interpeak variability |
| Faust 2020[4] | Computer-based (online for Main Study; disk-based for Pilot Study), home/on-site | Visual training (InSight software): Track moving targets, detect peripheral targets, spatial memory, fast motion direction identification Auditory training (Brain Fitness software): Tone discrimination, phoneme discrimination, syllable sequence recall, speech comprehension, sound-location matching | Waitlist control | Main Study: 8–10 weeks; 30–40 sessions (40 mins/session); 38 sessions total Pilot Study: 8–10 weeks; ≥30 sessions (30–40 mins/session); ≥900 mins total | main: 25.3; Pilot: ≥15 | Pre-test, post-test, and a three-month follow-up | None | RBANS Verbal Memory Index, Visual Processing Index, Working Memory Index, Processing Speed Index, and Total Scale Score | GDS |
| Fisher 2025 [5, 6] | Computer-based, home-based; provided calibrated headphones and laptop; Zoom-assisted setup | Adaptive training (APEX 4 software) with 6 real-world scenarios (weather, airport, restaurant, driving directions, medical appointment, shopping): Low demand – recall 2 keywords from the current target speaker’s sentence (multiple-choice); High demand – recall 2 keywords from the previous sentence | None | 10 sessions; 30 mins/session; 3 sessions/week; no more than 1 session/day | 5 | Pre-training and post-training | Unattended speaker intensity, SRT; Far-transfer task: SIN; SFG | Reading Span | NFC; SIM; TOI; Exit interview survey (training engagement, strategies, motivation ratings); Subjective listening effort ratings; MEG (temporal response function framework) |
| Frei 2025[7] | AU: Computer-based, Insert headphones; IM: Same as AU + HP Reverb G2 VR headset; Both: Laboratory-based | Adaptive AC training with natural continuous speech: 1. C component: Varying memory load (low: 1-sentence items; high: 2-sentence items) 2. Modality differences: AU-Auditory-only speech presentation + visual comprehension questions; IM-3D immersive audio-visual speech + visual comprehension questions | None | Two training sessions (AU + IM); 1 week apart; 40–45 mins/session. Each session: 60 items (30 low/30 high memory load) | 1.5 | Pre-training (before each training session) and post-training (after each training session) | SRT, PTA (0.5-4Hz) | MoCA, Working memory: Computerized n-back task in auditory and visual modalities | None |
| Frost 2025[8] | Mobile application-based (HELIX), home-based | Working and immediate memory: 1. Auditory task: Identify items under auditory maskers in different scenarios. 2. Recall task: Select images that represented the key words in the auditory instruction from the previous task. | None | 10 min per day, for 6 days per week for 4 weeks | 4 | Baseline, 4, and 8 weeks later | SHOEBOX online | ACE-III; DTT | 1. Quality of life questionnaires. 2. Paired semi-structured interviews and usability measures |
| Ferguson 2014 [9] | Computer-based, home-based | IHR-STAR platform "Phonomena" package: 11 phoneme continua; adaptive oddball paradigm; feedback-provided | No training during waitlist period (weeks 1–4); later received same ACT as IT group | 4 weeks; 15 min/day; 6 days/week; total 6 hours | 6 | Pre-training, post-training, 4-week retention, DT additional 12-week follow-up | PTA (0.25–8 kHz); SIN-ASL, DTT; Phoneme discrimination threshold (probe: /e/-/a/ continuum); Self-report-hearing: GHABP，SSQHS | Nonverbal IQ (Matrix Reasoning, WAIS); Working memory: Digit Span (WAIS-III), Visual Letter Monitoring (VLM); Divided attention: Test of Everyday Attention (TEA) subtests 6–7 (single/dual task) | None |
| Gundmi A , 2026 [10] | Clinic-based laptop-delivered training; supervised by audiologists in quiet test room | Culturally & linguistically Kannada-adapted LACE-derived computer training. Three core modules: degraded speech perception, auditory cognitive processing tasks, communication compensatory strategies. | Conventional aural rehabilitation: hearing aid fitting + one-off communication strategy counselling | 4 weeks total; 5 sessions per week, 60 minutes per single session (20 total sessions) | 20 | Baseline pre-intervention, post 4-week training | Quick-SIN; CST; LFT, HHIA | Reaction time | None |
| Henderson 2007 [11] | Computer-based, home-based; dedicated speakers or prototype hardware; adaptive difficulty (adjusted by response accuracy) | LACE software: 1. Degraded speech: SB, CS, TC speech; 2. C skills: TW, recall context words, MW, fill-in gaps; 3. Communication strategies: Environmental/counseling tips | No training during waitlist period (1 month) | 4 weeks; 30 min/day; 5 days/week; total 10 hours | 10 | Baseline, mid-training (2 weeks), post-training (4 weeks), and 8 weeks post-training; | PTA 0.5–2 kHz; QuickSIN (45 dB HL, 70 dB HL); HINT; Monosyllabic WRS; Self-report-hearing: HHIE/HHIA, CSOA: (strategies/attitudes subscales); | A memory (TW, MW tasks); speed of processing (TC task) | On-task training scores: SB, TC, CS, TW, MW |
| Kawata 2022[12] | In-person, soundproof rooms; supervised; audio stimuli via headphones | AC training: 3 tasks (short-term memory/word recall, working memory/number reverse recall, attention/go-no-go task); A factor (sound intensity level: SRT±3 to SRT-12 dB) + C factor (task difficulty: 1-4 levels); adaptive adjustment based on performance | Three C training tasks (same as training groups) at fixed level 1 (no variation in A/C difficulty) | 4 weeks; 2 days/week; 1 hour/session; total 8 sessions | 8 | pre-training, post-training, and 3 months follow-up | SRT, PTA (0.5-4Hz) | D-CAT, attention; LM-episodic memory; DS-working memory; MMSE | MRI: rGMV; resting-state FC |
| Kucuk Ceyhan 2022 [13] | Self-administered web-based computer training | LACE web-based adaptive training: speech-in-noise, rapid speech, competing speaker, auditory word memory tasks | Active control: 30-min daily short audio clips with simple comprehension questions; No-training control: no structured daily tasks | 11 sessions over 15 days, 30 mins per session, 1 session daily with 4 rest days | 5.5 | pre-training baseline, post-training immediately after 8-week intervention | QuickSIN test | WJ III short-term memory subtests; TEA attention subtests; One-Back & Number-Stroop dual tasks; TONI nonverbal reasoning | None |
| Lai 2023 [14] | Home DVD player self-administered adaptive auditory training with printed exercise sheets | LACE DVD training: speech-in-noise, rapid compressed speech, competing speaker sentences, cognitive word memory tasks + communication strategy guidance. | New control group: hearing aid use only, no training during 4-week trial | 4 weeks total; 10 lessons / 2 weeks, repeat all lessons for another 2 weeks; daily 30min session | 20 | Pre-training baseline; Post-training 1–2 weeks after training completion | QuickSIN (SNR loss); Compressed speech percent correct; SSI competing sentence correct; real-ear Verifit HA fitting verification; SSQ Speech & Other Qualities subscales | None | IOI-AI (training benefit questionnaire); IOI-HA |
| Olson 2013 [15] | Self-administered web-based computer training | LACE web-based adaptive training: speech-in-noise, rapid speech, competing speaker, auditory word memory tasks | Active control: 30-min daily short audio clips with simple comprehension questions; No-training control: no structured daily tasks | 11 sessions over 15 days, 30 mins per session, 1 session daily with 4 rest days | 5.5 | baseline, 2wk mid-training, 4wk post-training; control: baseline + 4wk only; no long-term delayed follow-up | QuickSIN test | WJ III short-term memory subtests; TEA attention subtests; One-Back & Number-Stroop dual tasks; TONI nonverbal reasoning | None |
| Ockelmann 2025 [16] | Mobile application-based (ActivEar, CogniFit), home-based | ActivEar training module: based on CogniFit's validated C training framework. ActivEar comprises a tailored subset of original CogniFit training games, supplemented by proprietary tasks specifically developed to target AC functions. | Foreign language training: The training was conducted in the computer-based language learning software Duolingo | At least 20 minutes a day, five days a week for 4 weeks | 6.7 | Pre-training, post-training, and a two-month follow-up | PTA (0.5-4Hz); SIN (Naturalistic speech-in-noise comprehension paradigm); OLSA; SSQHS | MoCA; 1. Verbal working memory: 2. Phonological short-term memory: FDS 3. Selective attention: VST 3. Divided attention: Visual dual task paradigm (DT) | EEG |
| Rao et al., 2017 | Laptop-based home audiovisual training, supervised lab sessions for first week; wear fitted HA during all practice | RMQ, audiovisual adaptive training: noise embedded witty sentence puzzles, combines speech perception + visual cue integration, auditory selective attention & memory demands | 30min daily audiobook listening with simple comprehension check questions (no adaptive speech training) | 4 weeks training phase, 5 days/week, minimum 30 mins daily | T group: 15.75; C group: 14.6 | Pre-HA fitting; 4 weeks post-HA acclimatization (pre-training); post 4-week training; no long-term follow-up | HINT SNR-50 SIN threshold; PTA | selective attention oddball task (d′ sensitivity, response criterion c, reaction time) | ERP electrophysiology (P3a, P3b amplitude/latency from three-stimulus oddball paradigm) |
| Rishiq 2016 [17] | Computer-based home training; supervised lab sessions in Week 1, self-led home sessions afterward | RMQ, adaptive audiovisual speech training: noisy audiovisual witty quip puzzle tasks, adaptive SNR adjustment, integrates auditory perception + visual speech reading | Wear binaural hearing aids daily without any structured auditory training | 4 weeks total, minimum 30 mins/day, 5 days per week | Minimum required 10 hours; average actual training time = 956.57 mins | HA fitting baseline (Pretest); 4 weeks HA acclimatization (Posttest I); additional 4 weeks training (Posttest II) | MLST-A: auditory-only (AO) & audiovisual (AV) speech recognition at -5 / 0 / +5 dB SNR | None | Real-ear HA fitting verification; hearing aid daily wear data logging |
| Saunders 2016 and Smith et al., 2016 [18, 19] | LACE-DVD: Home DVD player; LACE-C: Home laptop computer | LACE training modules: speech-in-noise, rapid compressed speech, competing speaker perception, auditory word memory, linguistic contextual reasoning + communication strategy guidance | Placebo: Passive audiobook listening with simple comprehension questions Standard control: one-on-one hearing loss educational counseling, no listening training | LACE-DVD: 10 sessions ×30 mins over 2 weeks LACE-C: 20 sessions ×30 mins over 4 weeks Placebo:20 sessions ×30 mins, 4 weeks Standard control: once, 30-min | LACE-DVD: 5; LACE-C: 10; Placebo:10; Standard control: 0.5 | 3 timepoints: baseline (V2); immediate post-intervention (V3); 6-month delayed follow-up (V4) | WIN; 45% & 65% time-compressed NU-6 speech; NU20 competing speech test | digit Span forward & backward (auditory short/working memory); multi-SPIN linguistic context utilization task | APHAP; HHIE; real-ear hearing aid verification |
| Whitton 2017 [20] | Home self-administered Microsoft Surface tablet softwar | Closed-loop (CL) audiomotor training: discriminate tone level/frequency/modulation in rising speech babble noise | WM adaptive auditory working memory puzzle training | 8 weeks total; 3.5 training hours per week, minimum 30 mins / single session | CL group average =35 hours; WM group average =31 hours | 3 test timepoints: Baseline, post-8week training, 7-week delayed maintenance follow-up | Monosyllabic WIN; QuickSiN; BKBSiN; competing multi-talker digit streaming task; FM detection threshold | Letter-Number Sequencing (WM capacity); Audio/Visual Stroop inhibitory control task | GDS, MoCA, IQ screening, training immersion/benefit expectation questionnaires; audio tracing error (CL on-task metric) |
| Yusof 2019 [21] | Computer-based, home-based, adaptive difficulty | Newly AC training system: 1. Word/sentence in noise (65 dB, 7 SNR levels): Select corresponding pictures after listening; 2. Word span/order/position: Select matching pictures for 2–9-word audio strings. | Watch documentary videos | 45 minutes/session, 3 sessions/week, 8 weeks, a total of 24 sessions, 18 hours | 18 | Baseline, and week 4, 8 and 12 | MyHIN, GIN, PPST | MoCA; DDT; | None |
| Zhao 2026 [22] | Home web/tablet self-administered gamified platform | Web-based gamified training: 1. Auditory module: speech-in-noise, competing talker, rapid speech comprehension 2. Cognitive module: executive function, visual-spatial skill, verbal/number memory, Chinese idiom dual processing | No training during 12-week trial | 12 weeks total; 5 sessions/week, 60 mins/session | Prescribed: 60 | Baseline (T0), Week 6 mid-assessment (T1), Week 12 post-intervention (T2) | PTA; Chinese HHIE-S | HK-MoCA; Chinese AVLT; TMT-A (focused attention), TMT-B (divided attention) | Social engagement: Lubben Social Network Scale; De Jong Gierveld Loneliness Scale; semi-structured qualitative acceptability interviews |
| Note: A, auditory; ABR, auditory brainstem response; ACE-III, Addenbrooke’s Cognitive Examination III; AC, auditory–cognitive; ACT, auditory–cognitive training; APEX, Audiological Platform for Experimental Research; ASL, adaptive sentence list; AU, audio-only group; C, cognitive; CS, competing speaker; CSOA, Communication Scale for Older Adults; D-CAT, digit-cancelation test; DS, digit span; DT, delay training; DTT, digit triplets test; EEG, electroencephalography; F₀ADD, fundamental frequency addition; FDS, forward digit span; FFR, frequency-following response; GDS, Geriatric Depression Scale; GHABP, Glasgow Hearing Aid Benefit Profile; GIN, gaps-in-noise test; H₂ADD, second harmonic addition; H₃SUB–H₆SUB, third- to sixth-harmonic subtraction; HHIA/HHIE, Hearing Handicap Inventory for Adults/Elderly; HINT, Hearing in Noise Test; HP Reverb G2 VR, HP Inc. Generation 2 virtual reality headset; IM, integrated multimodal group; LM, logical memory; MEG, magnetoencephalography; MLST, Multimodal Lexical Sentence Test; MMSE, Mini-Mental State Examination; MoCA, Montreal Cognitive Assessment; MW, missing word; MyHINT, Malay Hearing in Noise Test; NFC, Need for Cognition Scale; OLSA, Oldenburg sentence test; PPST, pitch pattern sequence test; PTA, pure-tone average; QuickSIN, Quick Speech-in-Noise test; RBANS, Repeatable Battery for the Assessment of Neuropsychological Status; RMQ, ReadMyQuips; SB, speech-in-babble; SFG, superior frontal gyrus; SIM, Situational Motivation Scale; SIN, speech in noise; SNR, signal-to-noise ratio; SRT, speech recognition threshold; SSQHS, Speech, Spatial and Qualities of Hearing Scale; TC, time-compressed; TFS, temporal fine structure; TOI; TW, target word; VST, Victoria Stroop Color-Word Test; WM, Word Memory; WJ-III, Woodcock–Johnson III Tests of Cognitive Abilities; WRS, monosyllabic word recognition score. | | | | | | | | | |

Table S2 Detailed Outcomes of Included Studies

| **Study ID** | **Key Findings** | **Task Effect** | **Time effect** | **Auditory related outcomes effect** | **Cognitive related outcomes effect** | **Neural Correlates Effect** |
| --- | --- | --- | --- | --- | --- | --- |
| Abrams et al., 2015 [1] | 1. Both groups demonstrated improved speech-in-noise performance on HINT and WIN after the intervention, but no statistically significant between-group differences in post-training improvement magnitude. 2. Cumulative training time significantly predicted post-intervention WIN scores but not HINT scores: more training hours correlated with lower SNR thresholds for 50% word recognition (r = -0.588, p = 0.001). | On-task effect：Not reported Off-task effect：Both groups showed mild speech-in-noise improvements with no additional benefit unique to the AT group; training hours were significantly associated with better WIN performance. | Short-term effect：Both groups showed mild speech-in-noise improvements with no additional benefit unique to the AT group; training hours were significantly associated with better WIN performance. Maintenance effect：Not reported | WIN (AT group only): Longer RMQ training hours correlated with better word-in-noise recognition (r = -0.588, p = 0.001). HINT: No significant group, condition, interaction or training hour effects. WIN between-group difference: No meaningful gap in post-training gains between AT and control. Hearing aid wear time: AT group’s milder wear-time decline was statistically insignificant. Aided listening benefit: Training produced no extra significant amplification advantage. | None | None |
| Anderson 2013 [2] | 1. Neurophysiology: ACT reduced envelope coding in hearing loss subgroup; no TFS changes;  2. Behavioral: ACT improved speech-in-noise perception, attention, and auditory short-term memory;  3. Control group: No significant changes. | On-task: Not assessed  Off-task: Generalization effects to speech-in-noise perception (specific to hearing-impaired group), attention (both hearing groups), and auditory short-term memory (specific to normal-hearing group); no corresponding changes in the active control group. | Short-term: 8-week training improved neurophysiological (normalized envelope encoding) and behavioral (QuickSIN, attention, memory) outcomes.  Long-term: Not reported | SINP (QuickSIN): Hearing-impaired group: 1.22 dB SNR loss reduction (~10% word recognition gain); normal-hearing group: no change (ceiling effect). | Sustained attention: Improved in both subgroups (auditory training group); no control group change. Auditory short-term memory: Improved only in the normal-hearing subgroup; hearing-impaired subgroup: no change (peripheral hearing limitation). | FFR: Hearing-impaired group: reduced envelope-dominated amplitudes; TFS-dominated amplitudes: no change.  ABR Wave V latency: Baseline-matched (no interaural difference ≥0.2 ms); no post-training changes.  Subcortical neuroplasticity: Training reversed imbalanced speech cue encoding via top-down modulation. |
| Anderson 2013 b [3] | 1. Neural: ACT Faster subcortical neural timing & reduced brainstem response peak variability (decreased temporal jitter) to speech signals; Behavioral: ACT Improved speech-in-noise perception, short-term memory, and C processing speed;  3. Control group: No significant changes in neural or behavioral outcomes. | On-task: Not assessed  Off-task: Generalization to speech-in-noise perception, auditory short-term memory, and visual processing speed (all improved in the auditory training group, p<0.05); no corresponding changes in the active control group (all p>0.15). | Short-term: 8-week training improved behavioral (QuickSIN, memory, processing speed) and neural outcomes.  Long-term: No follow-up data | SINP (QuickSIN): Auditory training group showed 0.69 dB SNR loss reduction; active control group had no significant change. | Auditory short-term memory: Auditory training group improved; active control group no change. Visual processing speed: Auditory training group improved; active control group no change. | ABR: Wave V latency baseline-matched; no post-training changes.  FFR timing: Auditory training group showed earlier peak latencies (34/44/54/64 ms) in noise (p<0.001) and quiet (p=0.025); reduced interpeak variability in noise (p=0.010). Quiet-to-noise lag: Auditory training group had smaller noise-induced timing shifts; active control group: no change. |
| Faust 2020[4] | Visual training yielded modality-specific transfer effects in both samples, transfer to working memory in the main sample, and transfer to processing speed in the pilot sample. There were no comparable transfer effects for auditory training. Combined-visual-and-auditory training failed to yield synergistic effects or any significant transfer effects. Visual processing transfer effects remained significant at follow-up. | On-task: Not assessed  Off-task: Visual games transferred to visual processing, working memory, and global cognition (pre-post); only visual processing transfer persisted at follow-up. Auditory games: No significant off-task transfer. Combined games: No significant off-task transfer effects. | Short-term: Visual games improved visual processing, working memory, and global cognition; auditory games only improved global cognition and processing speed (no control-adjusted transfer).  Long-term (3-month follow-up): Only visual processing remained significant; no other durable effects from auditory or combined games. | Not assessed | Visual processing: Visual games improved pre-post and maintained at follow-up; auditory games showed marginal improvement at follow-up.  Working memory: Visual games improved pre-post; auditory games had marginal pre-post and follow-up improvement.  Processing speed: No significant transfer.  Global cognition: Visual games improved pre-post; no follow-up effect.  Verbal memory: No significant improvement across all groups | Not assessed. |
| Fisher 2025 [5, 6] | Both low and high C demand AC training improved older adults’ SIN, working memory, and SFG, with reduced listening effort (neural and subjective); high-demand training led to poor performance and disengagement in some participants. Training enhanced neural contrast between attended and unattended stimuli, with pre-training SFG performance predicting this neuroplasticity. | On-task: Not assessed  Off-task: Both groups showed significant far-transfer improvements in speech-in-noise perception. No significant difference in transfer effects between the two cognitive demand groups. | Short-term: Improvements in SIN, working memory and nonlinguistic auditory stream segregation; reduced subjective listening effort. Neural changes included enhanced attentional contrast in 0 dB SNR condition (p=0.031) and increased M400 peak amplitude for attended speakers (p=0.006).  Long-term: No follow-up data reported | SINP: Significant pre-post improvement across both training groups in conditions with competing speakers (0 dB/6 dB SNR, babble).  SFG: Improved accuracy post-training (p=0.018); pre-training 8-chord SFG performance predicted neural plasticity (lower pre-training scores correlated with greater post-training attentional contrast).  PTA: No pre-post changes (all participants maintained ≤25 dB HL, 250–4000 Hz). | Working memory (reading span): Significant improvement post-training (F=15.79, p<0.001). | MEG measures: Post-training increased attentional contrast in speech envelope reconstruction (0 dB SNR condition, p=0.031); enhanced M400 peak amplitude for attended speakers (p=0.006) indicating improved intelligibility processing.  Neural prediction: Pre-training 8-chord SFG performance (25th/50th percentiles) predicted greater post-training attentional contrast (p<.05); 75th percentile performers showed no significant neural change. |
| Frei 2025[7] | Immersive ACT significantly improved speech-in-noise perception, with greater benefits for individuals with more pronounced hearing loss or reduced auditory working memory capacity. | On-task: Improved speech comprehension and memory for natural continuous speech in both AU and IM modalities. IM group showed better and more stable performancethan AU group.  Off-task: Generalized to speech-in-noise perception only after IM training; no significant transfer after AU training (Δβ=-0.118, p=0.817). | Short-term: Significant SRT reduction post-IM training; AU training showed minimal change (~0.12 dB). Modality × time interaction was significant.  Long-term: Not reported | SINP (OLSA test): Immersive (IM) training significantly reduced SRT; auditory-only (AU) training had no significant effect.  PTA: No pre-post changes after either training. | Visual working memory: No significant improvements.  Auditory working memory: Moderated the effect of IM training—participants with lower auditory working memory benefited more from IM training.  General cognitive function (MoCA): No significant changes. | Not assessed. |
| Frost 2025[8] | 1.An improvement in the performance of the Digit-Triplet-Test, measured 4 and 8 weeks after the baseline, was found within the training group; however, this improvement was not significant between the training and control groups. No significant improvements were found in any other outcome measures. 2.Thematic analysis suggested HELIX prompted the realization of difficulties and actions required, improved listening, and positive behaviour change. | On-task: The training focused on auditory tasks (identifying speech instructions in noise) and cognitive tasks (recalling key words in speech instructions). Results showed that the Signal-to-Noise Ratio (SNR) score of the TG significantly improved during the 4-week training period, indicating effective on-task learning.  Off-task: No significant off-task effect was observed. There were no statistically significant between-group differences in non-direct training-related indicators, including the DTT, battery of cognitive tests, Communication Confidence Profile (CCP), and WHO Quality of Life Age (WHOQOL-AGE) questionnaire (p>0.05 for all). | Short-term: After 4 weeks of intervention, the DTT score of the TG was significantly improved compared with the baseline; but this improvement was not statistically significant in the between-group comparison (mixed ANOVA); the on-task SNR score was significantly increased.  Long-term: the DTT score of the TG remained improved four weeks after the intervention | Significantly improved indicators:  1. On-task SNR: The score of the TG was significantly increased during training  2. DTT: Within the TG, the score was significantly improved from baseline to Week 4 and from baseline to Week 8.  Non-significantly improved indicators:  1. DTT: No significant between-group difference (mixed ANOVA ) | No significant improvement was observed in all cognitive test battery indicators, including Prospective Memory Word List, Target Detection, Selective Attention, 2D Manipulations, Blocks and Tower of London, Spatial Span, Choice Reaction Time, and Verbal Analogies. There were no statistically significant differences within the TG or between the two groups (p>0.05 for all) | None |
| Ferguson 2014 [9] | 1. On-task: Robust phoneme discrimination improvement, largest gains for poorest initial thresholds;  2. C: Improved divided attention and visual working memory; no improvement in simple C tasks;  3. Self-report: Reduced hearing disability, especially in group conversation;  4. Retention: Benefits maintained for 4 weeks post-training; no speech-in-noise perception improvement | On-task: Both groups showed significant training effects on the phoneme discrimination  Off-task: Among non-training-related indicators, complex cognitive tasks (divided attention, Visual Letter Monitoring), and self-reported hearing disability scores improved significantly; speech perception in noise (ASL, DTT) and simple cognitive tasks (single attention, DS) showed no significant improvement, indicating selective learning transfer. | Short-term: After 4 weeks of intervention, phoneme discrimination thresholds decreased significantly in both IT group and DT group; the IT group showed significant improvements in GHABP Disability score, TEA DTD, VLM.  Long-term：Four weeks after training (IT group t3, DT group t4), the improved effects of phoneme discrimination, divided attention, Visual Letter Monitoring, and self-reported hearing disability showed no significant attenuation, with a maintenance rate of 52%-75%, indicating stable training benefits. | Significantly improved indicators：  1. Phoneme discrimination threshold: All 11 phoneme continua decreased significantly, and the threshold of probe phoneme /a/-/e/ remained improved until follow-up in both groups.  Non-significantly improved indicators：  1. Speech perception in noise：ASL, SRT, DTT.  2. Subjective hearing scales：No significant improvement in any dimension of the SSQHS | Significantly improved indicators：  1. Divided attention：TEA DTD both decreased significantly  2. Visual working memory：VLM 1 letter/s and VLM 1 letter/2s both improved significantly.  Non-significantly improved indicators：  1. Single attention：No significant change in TEA single-task score.  2. DS：No significant improvement in working memory test. | None |
| Gundmi A et al., 2026[10] | 1. Significant Group × Time interaction for Quick-SIN (F=142.10, p<0.001): Intervention group average SNR improvement 7–8 dB, control only minimal ~0.4–1.0 dB gain; 85% trained participants reached ≥3dB clinically meaningful threshold. 2. Compressed & filtered speech perception showed significantly larger fold improvements in training arm. 3. Auditory processing speed: RTT and RTM reaction times significantly shortened in training group (F=93.46 & 77.88, p<0.001), no meaningful RT reduction in controls. 4. Self-reported hearing handicap (HHIA) reduced significantly for trained participants (F=81.04, p<0.001). | On-task: Not reported Off-task: Significant transfer to speech-in-noise, compressed/filtered speech, auditory processing speed and self-reported listening handicap. | Short-term: Large, consistent combined auditory-cognitive benefits unique to structured training group; control arm showed negligible practice-only small changes. Maintenance effect: Not reported | Quick-SIN SNR thresholds (7–8dB average reduction); all compressed & filtered speech recognition scores across degradation levels. Control group showed only trivial, non-clinically meaningful speech-in-noise changes. | Target word identification RT, missing word detection RT. | None |
| Henderson 2007 [11]  Sweet & Henderson Sabes, 2006[23] | 1. On-task: Significant improvement in SB (SNR reduced), CS (SNR reduced), TC (compression increased), TW/MW (difficulty elevated);  2. Generalization: Improved QuickSIN (45/70 dB) and HINT scores;  3. Self-report: Reduced hearing handicap and improved communication strategies/attitudes;  4. Predictors: Poorer baseline speech-in-noise performance, greater hearing loss, higher HHIE scores correlated with larger improvements;  5. Compliance: Older adults and those with greater hearing loss completed training on time/early; younger subjects had more scheduling conflicts. | On-task：All training tasks showed significant improvements, including SB, CS, TC, Auditory Memory, and MW. Significant differences were observed between training phases; greater improvements were seen in those with poorer initial performance.  Off-task：Standardized tests showed significant improvements, including QuickSIN at 45 dB, QuickSIN at 70 dB, HHIE, CSOA Strategy subscale , and CSOA Attitude subscale; the HINT showed numerical improvement but no statistical significance. | Short-term：After 4 weeks of intervention, all training tasks and off-task standardized tests (except HINT) showed significant improvements. Significant progress in tasks such as SB and CS was already observed at the mid-training (2 weeks).  Long-term：Not reported. | Significantly improved indicators：  1. Training tasks: Significant optimization of thresholds/difficulty in SB, CS, and TC tasks  2. Speech recognition in noise: QuickSIN 45 dB, QuickSIN 70 dB  3. Subjective hearing handicap: HHIE, CSOA Strategy subscale, CSOA Attitude subscale  Non-significantly improved indicators：  1. HINT: Numerical improvement but no statistical significance | Significantly improved indicators：  1. Auditory Memory: Significant increase in the difficulty level of training tasks  2. Information processing speed: Significant reduction in adjusted response time of MW tasks  3. TW task: Correlated with improvements in TC and MW tasks, suggesting involvement of working memory  Non-significantly improved indicators：None | None |
| Kucuk Ceyhan et al., 2022[13] | 1. Within the training group: Matrix test SNR thresholds significantly reduced (better noise speech recognition, p=0.004) and MMN mean latency significantly shortened (p=0.038) post-training; No significant pre-post changes observed in the control group for either metric. 2. MMN waveform detection rate rose from 76.48% to 94.12% in the training group after intervention; control group detection rate remained fixed at 56.25% at both time points. 3. No statistically significant between-group differences when comparing pre-post change magnitudes for MMN latency and Matrix SNR thresholds (all p>0.05). | On task: not reported; Off task: Speech in noise recognition and electrophysiological auditory discrimination MMN latency | Short-term: training group showed significant within-subject improvements in noise speech perception and MMN neural timing; control group had no significant changes. Maintenance effect: not reported. | Significantly improved indicators： Matrix test SNR threshold (within group, p = 0.004);MMN wave mean latency (within group, p=0.038);MMN detectability rate. Non-significantly improved indicators： Matrix test SNR threshold (between group);pre-post change for MMN latency between group | None | None |
| Lai et al., 2023[14] | 1. Only experimental group had significant pre-post QuickSIN SNR loss reduction; two control groups showed no meaningful auditory improvement. 2. On-task LACE metrics improved significantly within training group; competing speaker task no significant change. 3. All three groups showed general practice-related small gains across most cognitive tests (main effect of time), but no unique cognitive transfer benefit specific to LACE training. 4. No selective/sustained attention, short-term memory or nonverbal reasoning far-transfer improvements exclusive to training group. | On task: Significant within-group gains on LACE built-in speech-in-babble and word memory tasks; competing speaker task no change. Off task: Near transfer only to untrained QuickSIN speech-in-noise test; no cross-domain transfer to standardized memory, attention or nonverbal cognitive assessments. | Short-term: Auditory speech-in-noise benefit unique to LACE training group; universal mild practice gains on cognitive tasks for all three groups. Maintenance effect: not reported | Significantly improved indicators： QuickSIN SNR loss (only experimental group pre-post within-group improvement, significant group×time interaction p<0.001); LACE on-task speech-in-babble SNR & word memory scores (within training group). Non-significantly improved indicators： QuickSIN pre-post change in active control and no-training control groups; LACE competing speaker on-task performance | Significantly improved indicators：No Non-significantly improved indicators：short-term memory, selective/sustained attention, attentional switching or nonverbal reasoning | None |
| Kawata 2022[12] | 1. Behaviour: ATFGs improved PTA; C factor groups improved long-term memory and Digit-cancelation test; AC training showed no superior behavioral effects vs. single-modality training.  2. Neural: AC training increased rGMV in R. DLPFC, L. ITG, L. SFG, L. OFC, R. cerebellum (lobule 7 Crus 1); ATFGs increased L. TP rGMV and FC between L. TP and precuneus; CTFGs increased rGMV in R. IOG, R. cerebellum, L. ITG. | On-task: Not reported.  Off-task: Behavioral transfer: ATFGs (AC + A groups) improved auditory function (PTA); CTFGs (AC + C groups) enhanced episodic memory (LM) and attention (D-CAT); no cross-domain transfer effects (e.g., auditory training did not improve cognitive function, and vice versa). Neural transfer: the plasticity of the brain structure was observed after 4 weeks of training in the AC group | Short-term: Significant improvements in behavioral indicators (PTA, LM, D-CAT) and neural indicators (rGMV, FC) were observed after 4 weeks of training.  Long-term: No follow-up data reported; | PTA: ATFGs showed significant improvement compared to non-ATFGs. | Episodic memory (LM): CTFGs showed significant improvement compared to non-CTFGs.  Attention (D-CAT): CTFGs showed significant improvement compared to non-CTFGs.  Working memory (DS): No significant training effects across all groups.  General cognition (MMSE): No significant changes. | rGMV: AC group: Increased in the R. DLPFC, L. ITG, L. SFG, L. OFC, R. cerebellum (lobule 7 Crus 1).  ATFGs: Increased in the left temporal pole.  CTFGs: Increased in the right inferior occipital gyrus, right inferior temporal gyrus, and right cerebellum (lobule 7 Crus 1).  FC: ATFGs showed significantly enhanced positive FC between the left temporal pole and precuneus (FWE-corrected p<0.05). |
| Ockelmann 2025 [16] | 1. Participants completing ACT demonstrated significant improvements in SIN comprehension, with partial C gains, which remained at follow-up.  2. SIN intelligibility did not change in either group, indicating a dissociation between low-level fidelity and higher-order comprehension.  3. No changes emerged in subjective hearing reports. | On-task：Training targeted four cognitive domains: working memory, phonological short-term memory, selective attention, and divided attention. The experimental group showed significant improvements in all on-training cognitive tasks (working memory; phonological short-term memory; selective attention; divided attention).  Off-task：In lab-based untrained tasks, the experimental group showed significant improvements in phonological short-term memory, working memory, and selective attention (only divided attention showed no significant change); the control group showed significant improvement only in phonological short-term memory, with no significant changes in working memory and selective attention. Between-group differences indicated training-specific transfer effects. | Short-term：After 4 weeks of intervention, the experimental group demonstrated significantly better naturalistic SIN comprehension than the control group; all four on-task cognitive domains and lab-based working memory, phonological short-term memory, and selective attention were significantly improved; PTA, speech-in-noise intelligibility (OLSA), and subjective hearing score (SSQHS) showed no significant changes.  Long-term：At the 2-month follow-up, the improved effects of phonological short-term memory, selective attention, and working memory in the experimental group persisted; divided attention still showed no significant change; | Significantly improved indicators：  1. Naturalistic speech-in-noise (SIN) comprehension: The experimental group showed significant improvements under no-noise, low-noise (SNR=8dB), and high-noise (SNR=3dB) conditions, with significant between-group differences  Non-significantly improved indicators：  1. PTA: No significant changes from baseline to follow-up in both groups  2. SIN intelligibility (OLSA): No significant training effects in either group  3. Subjective hearing ability (SSQHS): No significant changes in either group  4. Subjective listening effort (NASA-TLX scale): No significant changes in either group | Significantly improved indicators：  1. On-training tasks: Working memory, phonological short-term memory, selective attention, and divided attention  2. Lab-based untrained tasks:  PSTM: Significantly improved in both groups;  WM: Significantly improved only in the experimental group;  FA: Significantly improved only in the experimental group;  3. Follow-up maintenance: Improved effects of phonological short-term memory, selective attention, and working memory persisted; selective attention further improved in the experimental group at follow-up  Non-significantly improved indicators：  Divided Attention (DA): No significant changes in on-training, lab-based tasks, or follow-up in either group | None |
| Olson et al., 2013 [15] | 1. Both new & experienced HA training groups showed significant speech-in-noise gains vs HA-only control; new users demonstrated larger effect sizes and faster improvements. 2. New users reported significantly higher perceived training benefit on IOI-AI than experienced users (p=0.018). 3. Training compliance high (94% new,98.5% experienced); minor DVD technical issues reported by 25% participants. 4. SSQ speech domain scores significantly improved from baseline to 4wk for all training participants. | On-task: Progressive adaptive difficulty improvements across all LACE training modules. Off-task: Near transfer to untrained QuickSIN, compressed speech, SSI competing speech; subjective real-world listening gains on SSQ. | Short-term: Significant speech perception improvements at 2 weeks training for new HA users; experienced users required full 4 weeks for meaningful gains. Maintenance effect: Not reported | Significantly improved indicators： QuickSIN SNR (new + experienced training vs new control); SSI competing sentence accuracy (training groups within-group); SSQ speech subscale scores; compressed speech recognition (within training groups). Non-significantly improved indicators： Between-group difference for compressed speech; no meaningful SSQ Other Qualities subscale change across all groups. | Not reported | None |
| Rao et al., 2017[24] | 1. Hearing aid use alone: No behavioral HINT/attention changes, but P3a amplitude significantly reduced. 2. Only experimental group showed significant HINT SNR-50 improvement (Group×Session interaction p<0.001). 3. Selective attention significantly reduced in training group. 4. RMQ group showed enhanced target-related cortical attention processing; P3a showed no additional training-induced shift after HA acclimatization. 5. Passive audiobook control showed no SIN or selective attention training-specific gains. | On-task: Not reported Off-task: Near transfer to HINT SIN; selective attention judgment criterion improvements specific to training group; no additional P3a neural plasticity beyond HA acclimatization period. | Short-term: HA wear alone induced neural P3a plasticity without behavioral speech gains; 4-week RMQ training yielded unique speech-in-noise and attention criterion improvements not seen in controls. Maintenance effect: Not reported | Significantly improved indicators： HINT SNR-50 (within experimental group pre vs post training, significant group×session interaction). Non-significantly improved indicators： HINT change for control group. | Significantly improved indicators： Selective attention response criterion c (only RMQ training group showed significant reduction post-training). Non-significantly improved indicators： d′ discriminability & reaction time: only universal practice effect across both groups, no training-specific unique gains. | Significantly improved neural indicators： P3a amplitude reduction after 4 weeks hearing aid acclimatization (all participants, linked to better attention sensitivity); post-training P3b amplitude change positively correlated with d′ improvement exclusively in RMQ group. Non-significant neural indicators： No further P3a amplitude/latency changes after RMQ training; P3b amplitude/latency showed no main group or session interaction effects across pre/post training. |
| Rishiq et al., 2016 [17] | 1. Significant main effects of presentation modality (AV > AO performance) and SNR were confirmed across all participants. 2. Significant Test × SNR interaction: all participants improved speech recognition at +5 dB SNR after 4 weeks of HA acclimatization, regardless of training. 3. No significant Group × Test interaction; RMQ training did not produce unique improvements in either auditory-only or audiovisual speech recognition on MLST-A. | On-task: Not reported Off-task: No significant far transfer to untrained fixed-SNR AO/AV speech recognition measured by MLST-A. | Short-term: speech recognition gains after HA use alone; no extra AV/AO gains from additional RMQ training. Maintenance effect: Not reported | Significantly improved indicators： All participants showed better AO/AV performance at +5 dB SNR after 4 weeks of hearing aid acclimatization (main effect of time); AV modality scores consistently superior to AO at all SNRs. Non-significantly improved indicators： No unique pre-post MLST-A improvements specific to RMQ training group (no group×time interaction across all SNR & modality conditions). | None | None |
| Saunders et al., 2016; [18]  Smith et al., 2016[19] | 1.No statistically significant between-group main effects or interactions for any behavioral/cognitive/subjective outcome, regardless of LACE format, hearing aid experience or follow-up time point. 2.Significant on-task improvements within LACE training software, but no generalized transfer to standardized untrained speech/cognitive tests. 3. Baseline poorer performance predicted small post-test gains across ALL four arms (training/placebo/control), meaning improvements reflected general practice effects rather than LACE-specific training benefit. 4.No group differences in HHI or daily listening activity limitations at post-training or 6-month follow-up. 5.Attrition rate only 12.9%; training compliance high | On-task: Significant within-program improvements observed for all LACE trained participants on built-in training tasks. Off-task: No significant cross-task generalization to SIN, rapid speech, competing speech, auditory memory or contextual processing assessments. | Short-term: No unique LACE-induced gains vs placebo/control Maintenance effect: No sustained training-specific behavioral or subjective benefits detected for either LACE format. | Significantly improved indicators： None Non-significantly improved indicators： WIN, compressed speech, competing speech performance for LACE-DVD / LACE-C groups relative to placebo/control at post-training or 6-month follow-up. | Significantly improved indicators： Universal mild practice shifts on digit span & linguistic context task. Non-significantly improved indicators： Auditory short-term/working memory and linguistic context utilization | None |
| Whitton et al., 2017 [20] | 1. Both groups showed comparable within-task learning gains and subjective expectation of hearing improvement (valid WM as matched placebo control). 2. Only CL audiomotor training produced generalized off-task SIN gains; no transfer to single-word recognition. 3. CL training benefit was 3× larger than hearing aid-only benefit at challenging low SNRs; 62% CL participants reached clinically meaningful speech improvement vs only 9% WM participants. 4. Better CL game tracing accuracy predicted larger SIN gains; higher baseline inhibitory control predicted greater training response. 5. WM training improved only on-task memory performance, without transfer to untrained speech or standardized WM tests. 6. All CL speech gains fully faded after 7 weeks of no ongoing practice. | On-task: CL group significantly reduced audio tracing error; WM group showed improved keyword recall capacity (both groups large within-task learning). Off-task: CL training transferred to untrained sentence/multi-digit stream SIN tasks; WM training showed no cross-task generalization to any speech or standardized cognitive measures. | Short-term: CL group showed large, statistically significant speech intelligibility gains at low SNRs; WM group no meaningful speech improvement. Maintenance effect: All CL speech transfer effects disappeared; no durable off-task benefit without continuous practice. | Significantly improved indicators： Low/high context sentence recognition accuracy (low SNR conditions); multi-talker digit sequence full recall; audio tracing performance within CL training tasks. Non-significant indicators： Isolated single-word WIN recognition; FM temporal fine structure detection thresholds; no speech gains in WM control group. | Significantly improved indicators： WM training’s in-game keyword recall on-task performance. Non-significant indicators： Letter-Number Sequencing working memory scores; audio/visual Stroop inhibitory control (no training-induced change for either group). | None |
| Yusof 2019 [21] | 1. The newly developed ACTS can produce training effects in speech recognition (in quiet), A processing and in cognition.  2.This study has also shown that the training effects were sustained at least up to 1 month post-training.  3. Training effects were seen in more domains in the normal cognition group than those in the Neurocognitive impairment group, suggesting a higher learning potential among the normal cognition participants. | On-task: The training focused on auditory-cognitive related tasks, including Word in Noise, Sentence in Noise, Word Span, Word Order, and Word Position. These tasks target speech-in-noise reception ability, working memory capacity, and cognitive interference suppression. Significant effects were observed on outcome indicators directly associated with these training tasks, such as speech recognition tests, central auditory processing assessments, and cognitive evaluations.  Off-task: No explicit reports on off-task effects were provided. The study did not investigate the impact of training on non-training-related domains, such as daily living activities, other sensory functions, or non-auditory/cognitive abilities. | Short-term：Significant training effects were detected in both groups. The NC group showed effects in Malay Hearing in HINT quiet, GIN, PPST (humming), DDT, Delayed Memory, and MoCA. While the NCI group exhibited significant effects in DDT, Delayed Memory, and MoCA.  Long-term: At Week 12 (4 weeks after the end of training), paired sample t-tests revealed no significant attenuation of training effects in either group. Specifically, the NC group maintained effects on the MyHINT (quiet condition), GIN Test, PPST (humming mode), DDT delayed memory, and total MoCA score. The NCI group maintained effects on the DDT, delayed memory, and total MoCA score. All training effects were sustained for at least 1 month post-training. | Significantly improved indicators：  1. NC group： MyHINT (quiet condition) , GIN Test, PPST, Malay DDT  2. NCI group：Malay DDT  Non-significantly improved indicators：  1. MyHINT (composite noise condition)：No significant training effects in either group.  2. Neurocognitive Impairment (NCI) group：GIN Test, PPST (humming response mode), PPST (verbal labeling response mode) | Significantly improved indicators：  1. Total MoCA score：both NC and NCI group improved.  2. Delayed memory domain of MoCA：both NC and NCI group improved.  Non-significantly improved indicators：No significant improvements were reported for other cognitive subdomains. The study only analyzed the total MoCA score and delayed memory domain, without investigating independent effects on other cognitive domains. | None |
| Zhao et al., 2026 [22] | 1. Cognitive benefits exclusive to training group: Significant within-group gains on TMT-A, TMT-B, HK-MoCA naming & visuospatial subdomains, AVLT 5-min delayed recall. Control only showed trivial practice-related cognitive shifts. 3. Both groups reported reduced self-perceived hearing difficulty; training group had larger effect on emotional hearing handicap. 4. PTA: Significant time×group interaction for left ear thresholds, no large peripheral hearing improvement overall. 5. No significant group differences in loneliness or social engagement scores. | On-task: Progressive adaptive difficulty improvements observed across all built-in auditory and cognitive gamified modules. Off-task: Significant far transfer to attention, visual cognition and verbal delayed memory tests; limited transfer to pure-tone peripheral hearing thresholds; no transfer to loneliness/social connection metrics. | Short-term: Training group elevated HK-MoCA total scores, cognitive gains sustained to week 12. Maintenance effect: Not reported | Significantly improved indicators： Left-ear PTA; emotional hearing handicap. Non-significantly improved indicators： Right-ear PTA; overall PTA between-group main effect absent; social subscale of HHIE. | Significantly improved indicators： TMT-A focused attention; TMT-B divided attention; HK-MoCA naming & visuospatial subscales; AVLT 5-minute delayed recall. Non-significantly improved indicators： HK-MoCA total global score between-group difference; AVLT recognition memory only trivial within-group shift; HK-MoCA memory/orientation/abstraction domains change for either arm. | None |
| Note: A, Auditory; C, Cognitive; AC, Auditory Cognitive; ACT, Auditory Cognitive Training; ACTS, Auditory Cognitive Training System; ABR, Auditory Brainstem Response; ACE-III, Addenbrooke’s Cognitive Examination III; APEX, Audiological Platform for Experimental Research; APHAP, Abbreviated Profile of Hearing Aid Benefit; ASL, Adaptive Sentence List; ATFGs, Auditory Factor Groups; AU, Audio-only Group; AVLT, Auditory Verbal Learning Test; CCP, Communication Confidence Profile; CL, Closed-Loop auditory training; CS, Competing Speaker; CSOA, Communication Scale for Older Adults; CTFGs, Cognitive Factor Groups; CST, Compressed Speech Test; D-CAT, Digit Cancellation Test; DA, Divided Attention; DDT, Dichotic Digit Test; DTD, Dual-Task Decrement; DS, Digit Span; DT, Delay Training; DTT, Digit Triplets Test; ERP, Event-Related Potential; FC, Functional Connectivity; FFR, Frequency-Following Response; F₀ADD, Fundamental Frequency Addition; FDS, Forward Digit Span; FM, Frequency Modulation; GDS, Geriatric Depression Scale; GHABP, Glasgow Hearing Aid Benefit Profile; GIN, Gaps in Noise; H₂ADD, Second Harmonic Addition; H₃SUB–H₆SUB, Third to Sixth Harmonic Subtraction; HHIA, Hearing Handicap Inventory for Adults; HHIE, Hearing Handicap Inventory for the Elderly; HINT, Hearing in Noise Test; HELIX, Auditory-Cognitive Training Program (HELIX); IM, Integrated Multimodal Group; IOI, International Outcome Inventory; IT, Immediate Training; L. ITG, Left Inferior Temporal Gyrus; L. OFC, Left Orbitofrontal Cortex; L. SFG, Left Superior Frontal Gyrus; L. TP, Left Temporal Pole; LFT, Low-pass Filtered Speech Test; LM, Logical Memory; M400, M400 Component; MEG, Magnetoencephalography; MLST, Multimodal Lexical Sentence Test; MMN, Mismatch Negativity; MoCA, Montreal Cognitive Assessment; MMSE, Mini-Mental State Examination; MW, Missing Word; MyHINT, Malay Hearing in Noise Test; NASA-TLX, NASA Task Load Index; NC, Normal Cognition; NCI, Neurocognitive Impairment; OLSA, Oldenburger Sentence Test; PSTM, Phonological Short-Term Memory; PTA, Pure-Tone Average; PPST, Pitch Pattern Sequence Test; RBANS, Repeatable Battery for the Assessment of Neuropsychological Status; RMQ, ReadMyQuips; rGMV, Regional Gray Matter Volume; R. DLPFC, Right Dorsolateral Prefrontal Cortex; SIN, Speech in Noise; SINP, Speech-in-Noise Perception; SNR, Signal-to-Noise Ratio; SSI, Synthetic Sentence Identification; SB, Speech-in-Babble; SUB, Subtraction; SSQHS, Speech, Spatial, and Qualities of Hearing Scale; SRT, Speech Recognition Threshold; TC, Time-Compressed; TEA, Test of Everyday Attention; TFS, Temporal Fine Structure; TG, Training Group; TMT, Turkish Matrix Test; TW, Target Word; VLM, Visual Letter Monitoring; VR, Virtual Reality; VST, Victoria Version of the Stroop Color-Word; WASI, Wechsler Abbreviated Scale of Intelligence; WHOQOL-AGE, World Health Organization Quality of Life in Old Age; WJ_III, Woodcock-Johnson III Tests of Cognitive Abilities; WIN, Words-in-Noise Test; QuickSIN, Quick Speech-in-Noise Test. | | | | | | |

Figure S1 Funnel plot for speech in noise

Figure S2 Subgroup analysis of ACT Effects on speech-in-noise performance by hearing aid Use

Figure S3 Subgroup analysis of ACT Effects on memory by domain

**References**

1.	Abrams, H.B., K. Bock, and R.L. Irey, *Can a Remotely Delivered Auditory Training Program Improve Speech-in-Noise Understanding?* Am J Audiol, 2015. **24**(3): p. 333-7.

2.	Anderson, S., et al., *Training changes processing of speech cues in older adults with hearing loss.* Front Syst Neurosci, 2013. **7**: p. 97.

3.	Anderson, S., et al., *Reversal of age-related neural timing delays with training.* Proceedings of the National Academy of Sciences of the United States of America, 2013. **110**(11): p. 4357-4362.

4.	Faust, M.E., et al., *Exploring the Specificity, Synergy, and Durability of Auditory and Visual Computer Gameplay Transfer Effects in Healthy Older Adults.* The journals of gerontology. Series B, Psychological sciences and social sciences, 2020. **75**(6): p. 1170-1180.

5.	Fisher, C., et al., *The Impacts of Two Adaptive Auditory-Cognitive Training Paradigms on Listening to Competing Talkers.* Seminars in Hearing, 2025. **46**(2): p. 141-160.

6.	Fisher, C., et al., *Neural and Behavioral Changes in Older Adults from Auditory-Cognitive Training.* Annual International Conference of the IEEE Engineering in Medicine and Biology Society. IEEE Engineering in Medicine and Biology Society. Annual International Conference, 2025. **2025**: p. 1-6.

7.	Frei, V. and N. Giroud, *Immersive auditory-cognitive training improves speech-in-noise perception in older adults with varying hearing and working memory.* NPJ Sci Learn, 2025. **10**(1): p. 12.

8.	Frost, E., et al., *HEaring and LIstening eXperience (HELIX): Evaluation of a co-designed serious game for auditory-cognitive training.* Q J Exp Psychol (Hove), 2025: p. 17470218251324168.

9.	Ferguson, M.A., et al., *Benefits of phoneme discrimination training in a randomized controlled trial of 50- to 74-year-olds with mild hearing loss.* Ear Hear, 2014. **35**(4): p. e110-21.

10.	Gundmi, A., et al., *Efficacy of a structured auditory training module in older individuals with hearing loss: a randomized controlled trial.* Eur Arch Otorhinolaryngol, 2026. **283**(6): p. 3585-3594.

11.	Henderson Sabes, J. and R.W. Sweetow, *Variables predicting outcomes on listening and communication enhancement (LACE) training.* Int J Audiol, 2007. **46**(7): p. 374-83.

12.	Kawata, N.Y.S., et al., *Auditory Cognitive Training Improves Brain Plasticity in Healthy Older Adults: Evidence From a Randomized Controlled Trial.* Front Aging Neurosci, 2022. **14**: p. 826672.

13.	Kucuk Ceyhan, A., H.H. Dere, and B. Mujdeci, *Evaluating the Effectiveness of a New Auditory Training Program on the Speech Recognition Skills and Auditory Event-Related Potentials in Elderly Hearing Aid Users.* Audiol Neurootol, 2022. **27**(5): p. 368-376.

14.	Lai, C.Y., et al., *Effects of Auditory Training in Older Adults.* J Speech Lang Hear Res, 2023. **66**(10): p. 4137-4149.

15.	Olson, A.D., J.E. Preminger, and J.B. Shinn, *The effect of LACE DVD training in new and experienced hearing aid users.* J Am Acad Audiol, 2013. **24**(3): p. 214-30.

16.	Ockelmann, J., et al., *Personalized and gamified auditory-cognitive training improves naturalistic speech-in-noise comprehension in older adults with hearing loss.* NPJ Sci Learn, 2025. **10**(1): p. 80.

17.	Rishiq, D., et al., *Can a Commercially Available Auditory Training Program Improve Audiovisual Speech Performance?* Am J Audiol, 2016. **25**(3s): p. 308-312.

18.	Saunders, G.H., et al., *A Randomized Control Trial: Supplementing Hearing Aid Use with Listening and Communication Enhancement (LACE) Auditory Training.* Ear Hear, 2016. **37**(4): p. 381-96.

19.	Smith, S.L., et al., *Examination of Individual Differences in Outcomes From a Randomized Controlled Clinical Trial Comparing Formal and Informal Individual Auditory Training Programs.* J Speech Lang Hear Res, 2016. **59**(4): p. 876-86.

20.	Whitton, J.P., et al., *Audiomotor Perceptual Training Enhances Speech Intelligibility in Background Noise.* Curr Biol, 2017. **27**(21): p. 3237-3247.e6.

21.	Yusof, Y., et al., *Efficacy of a newly developed auditory–cognitive training system on speech recognition, central auditory processing and cognitive ability among older adults with normal cognition and with neurocognitive impairment.* Geriatrics and Gerontology International, 2019. **19**(8): p. 768-773.

22.	Zhao, I.Y., et al., *Web-Based Gamified Auditory-Cognitive Dual-Task Training for Older Adults With Age-Related Hearing Loss: Pilot Randomized Controlled Trial.* JMIR Aging, 2026. **9**: p. e84083.

23.	Sweetow, R.W. and J.H. Sabes, *The need for and development of an adaptive Listening and Communication Enhancement (LACE) Program.* J Am Acad Audiol, 2006. **17**(8): p. 538-58.

24.	Rao, A., et al., *Neural Correlates of Selective Attention With Hearing Aid Use Followed by ReadMyQuips Auditory Training Program.* Ear Hear, 2017. **38**(1): p. 28-41.
